# CircRNA-5335 Regulates the Differentiation and Proliferation of Sheep Preadipocyte via the miR-125a-3p/STAT3 Pathway

**DOI:** 10.3390/vetsci11020070

**Published:** 2024-02-04

**Authors:** Wei Guo, Renzeng Ciwang, Lei Wang, Shuer Zhang, Nan Liu, Jinshan Zhao, Lisheng Zhou, Hegang Li, Xiaoxiao Gao, Jianning He

**Affiliations:** 1 College of Animal Science and Technology, Qingdao Agricultural University, Qingdao 266109, China; 2 Institute of Animal Science, Tibet Academy of Agricultural and Animal Husbandry Sciences, Lhasa 850009, China; 3 Shandong Animal Husbandry Chief Station, Jinan 250100, China

**Keywords:** sheep, preadipocyte, STAT3, miR-125a-3p, circRNA-5335

## Abstract

**Simple Summary:**

Mutton is a popular source of high-quality protein and nutrients. It is considered a green food due to its low cholesterol levels and high nutritional value. Adequate intramuscular fat may increase marbling, decrease shearing forces, and improve meat taste and palatability. Intramuscular white fat cells are produced by the differentiation of preadipocytes. The content of intramuscular fat is directly related to meat quality. The influence of preadipocytes on intramuscular fat content is closely connected with the proliferation and differentiation of preadipocytes. Therefore, the study of precursor fat cells is becoming increasingly more detailed. Studying the expression of the circRNA-5335/miR-125a-3p/STAT3 network at the cellular and molecular levels is crucial to enhance mutton quality by improving fat formation.

**Abstract:**

The content of intramuscular fat (IMF) from preadipocytes is proportional to meat quality in livestock. However, the roles of circRNAs in IMF deposition in sheep are not well known. In this study, we show that circRNA-5335/miR-125a-3p/STAT3 play a crucial adjective role in the proliferation and differentiation of sheep preadipocytes. In this study, we characterized the roles of differentially expressed circRNA-5335/miR-125a-3p/STAT3, which were screened from sheep of different months of age and based on sequencing data. Firstly, the expression profiles of circRNA-5335/miR-125a-3p/STAT3 were identified during the differentiation of preadipocytes in vitro by RT-qPCR and WB. Then, the targeting relationship of the circRNA-5335/miR-125a-3p/STAT3 was verified by dual-luciferase reporter assays. The results of RT-qPCR, CCK8, EdU and Oil Red O staining assay showed that miR-125a-3p suppressed the differentiation and raised the proliferation of preadipocytes by targeting STAT3. As a competing endogenous RNA, the downregulation of circRNA-5335 decreased the expression of STAT3 by increasing miR-125a-3p, which inhibited the differentiation of preadipocytes and promoted proliferation. Our present study demonstrates the functional significance of circRNA-5335/miR-125a-3p/STAT3 in the differentiation of sheep preadipocytes, and provides novel insights into exploring the mechanism of IMF.

## 1. Introduction

### 1.1. Intramuscular Fat Accumulation and Preadipocytes in Sheep

The content of intramuscular fat (IMF) is recognized as the most important factor influencing the quality of meat. [1]. The IMF is located between muscle fibers and bundles. Different amounts and distributions of IMF may result in different degrees of marbling in muscles, which can affect meat flavor and palatability [2,3]. Previous studies indicated that mutton juiciness decreased with the reduction in IMF content [4,5]. The mesenchymal progenitor cells generated from the precursors of pluripotent stem cells differentiate into preadipocytes and then mature into adipocytes, which ultimately affects IMF content [6]. However, the molecular mechanisms of adipogenic differentiation in sheep remain poorly evaluated.

### 1.2. IMF Regulatory Factors and Pathways

The deposition of IMF is regulated by a huge number of factors, including breed, genetic factors, age and diet. [7]. Researchers indicated that the estimated heritability of IMF content is more than 0.45 in sheep, indicating that we could improve the quality of IMF content through genetic selection. Multiple genes involved in fat metabolism participate in the deposition of IMF [8,9]. The initial phase (4d) of adipose differentiation involves the activation of the signal transducer and activator of transcription-3 (STAT3) protein [10]. The Janus kinase (JAK)/STAT3 pathway manages the early stages of differentiation through C/EBP-β transcription [11]. Furthermore, the activation of STAT3 by C/EBP-β and C/EBP-δ regulates the expression of CCAAT enhancer binding protein alpha (C/EBPA) and peroxisome proliferator-activated receptor gamma (PPARG), indicating that STAT3 is a regulatory target in the differentiation of fat cells and affects the activation of PPARG [12]. The effects of STAT3 on fat deposition by affecting levels of PPARG and C/EBPA have been well documented [6]. Nevertheless, the mechanism of STAT3 in regulating both the proliferation and differentiation of sheep preadipocytes is unclear.

### 1.3. CircRNA and IMF

CircRNAs, a class of non-coding RNAs (ncRNAs), are produced by the reverse splicing of mRNA precursors. In recent years, the rapid development of RNA-Seq has greatly promoted the discovery of circRNAs [13]. Commonly, circRNA molecules, as competitive endogenous RNAs (ceRNAs), can influence gene splicing, transcription and the modification of parental gene expression [14]. To date, a number of circRNAs have been identified as novel regulators that regulate cell biological processes in fat formation. For example, circFUT10 plays a role in promoting the proliferation of preadipocytes [15]. The overexpression of circFLT1 and lncCCPG1 promotes preadipocyte differentiation, but inhibits their proliferation [16]. There are many studies on miRNAs showing their regulatory effects on preadipocyte proliferation and differentiation in different species. For example, the overexpression of miR-125a-5p promotes the proliferation but inhibits the differentiation of preadipocytes in pigs [17], and miR-125a-3p may regulate preadipocyte differentiation by targeting FSTL1 [18]. Furthermore, previous research has investigated the effect of circHOMER1 on porcine adipogenesis ex vivo and in vivo, demonstrating that circHOMER1 performed a depressant effect in adipogenesis by SIRT1 and miR-23b [19].

Sheep are an extremely valuable species that can supply fine wool and high-quality meat, but the molecular mechanism of IMF deposition remains largely unknown. Here, we examined the circRNA-5335/miR-125a-3p/STAT3 pathway, which was screened from the longissimus dorsi muscle of sheep [20,21], to understand its regulatory role on IMF and examine the targeting relationship between circRNA-5335 and miR-125a-3p and its effect on both the proliferation and differentiation of preadipocytes. Studying the expression of the circRNA-5335/miR-125a-3p/STAT3 network at the cellular and molecular levels is crucial to enhance mutton quality by improving fat formation. Molecular markers related to intramuscular fat are of great importance in the promotion of the breeding of new breeds of high-quality sheep.

## 2. Materials and Methods

### 2.1. Cell Separation and Culture

The intramuscular preadipocytes obtained in this study were taken from a 3-day-old ram which was anesthetized [22]. The lamb was bled to death, and about 10 g of the longissimus dorsi muscle was removed. The obtained muscle was washed 3–5 times using PBS (SH30256.01, hyclone, Logan, UT, USA), followed by a quick wash with 75% absolute ethanol and then again 3–5 times with PBS. After removing impurities from the dorsal muscle under a stereoscope, the muscle was cut into about 1 g small pieces, which were homogenized into cell suspension using two volumes of 0.2% collagenase Ⅱ (C8150, Solarbio, Beijing, China). The cell fractions that had been digested were centrifuged at 1000 rpm for 5 min. After being sieved into a new centrifuge tube, the cells underwent centrifugation. Subsequently, after centrifugation, the supernatant was discarded and then the cells were resuspended and stored [23].

The cell culture medium was 90% Dulbecco’s modified Eagle medium/F12(DMEM/F12, SH30023.01, hyclone, Logan, UT, USA) supplemented with 10% fetal bovine serum (FBS, 10099-141, Gibco, Grand Island, NY, USA) and 5% penicillin–streptomycin (P/S, SV30010, hyclone, Logan, UT, USA). The cells were cultured overnight at 5% CO_2_, 37 °C and 95% humidity [24].

### 2.2. Dual-Luciferase Activity Assay

For plasmid synthesis, the targeted fragments of miR-125a-3p and STAT3 and of circRNA-5335 and miR-125a-3p were selected and the two targeted fragments were mutated. Four of the sequences were submitted to Tsingke Biotechnology (Beijing, China) for the synthesis of gene fragments and PmirGLO plasmid. To examine the targeted relationship between circRNA and miRNA as well as the target gene, plasmids were constructed with corresponding sequences and transfected into 293T cells in groups using the transfection reagent Invitrogen™ Lipofectamine™ 3000 (L3000015, Thermo Fisher, Waltham, MA, USA). Then, the fluorescence in 293T cells was detected by a Dual-Luciferase Reporter Assay Kit (DL101-01, vazyme, Nanjing, China) on a luminometer (GLOMAX-20/20, Promega, Madison, WI, USA). The ratio of two fluorescence values in the experimental group and the control group was estimated using a fluorescence detector to analyze the target binding [25].

### 2.3. Identification of circRNA

The binding sites between circRNA and miRNA can be predicted from their sequences. CircRNA acts as a sponge for the target miRNA. We extracted gDNA from the longissimus dorsi muscle tissue. The total RNA was extracted using TRIzol (9108, Takara, Shiga, Japan); the linear RNA was eliminated from the RNA substrate by Ribonuclease R (RNase R, R0301, Geneseed, Guangzhou, China), and was converted to cDNA using a reverse transcription kit (R312-01, Vazyme, Nanjing, China). The divergent and convergent primers were designed in order to check whether the gDNA and cDNA met the cyclization requirement by electrophoresis.

### 2.4. Preadipocyte Differentiation 

The cells were subcultured into 6-well plates and differentiated in stages, and transfection was performed when the density of the cells reached 70%. For the first 2 days, the preadipocytes were cultivated by 0.5 mmol/L of 3-isobutyl-1-methylxanthine (IBMX, I7018, Sigma, St. Louis, MO, USA), 1umol/L of dexamethasone (DEX, D4902, Sigma, St. Louis, MO, USA) and 10 µg/mL of insulin (INS, I8830, Solarbio, Beijing, China) with culture medium. Two days later, 10 µg/mL of INS was added to the culture cells. The culture medium was changed every 2 days and this process continued until the 8th day [23]. 

### 2.5. Oil Red O(ORO) Staining

To confirm the cells’ differentiation into adipocytes, ORO staining of the lipid droplets was performed. The process of induced differentiation was carried out after transfection. The cells were stained using an Oil Red O Stain Kit (G1262, Solarbio, Beijing, China) after induced differentiation, and the degree of cell staining was compared to understand the influence of the RNA transfected into the cells [20].

### 2.6. Cell Proliferation Assay

After transfecting the corresponding RNA fragments into the respective group of cells, Cell Counting Kit 8(CCK8, 40203ES76, Beyotime, Nanjing, China) reagents were added to the cells. The mixture’s absorbance at 450 nm was determined by a continuous-wavelength labeling instrument (Infinite M Nano, Tecan, Seoul, Republic of Korea) after an appropriate incubation time [26]. Then, 5-ethynyl-2′-deoxyuridine (EdU) analysis was performed on the cells using a BeyoClick™ EdU Cell Proliferation Kit with Alexa Fluor 488 (C0071S, Beyotime, Nanjing, China). The proliferation analysis was carried out using the Axio scope A1 microscope (ZEISS, Oberkochen, Germany).

### 2.7. Real-Time Quantitative PCR (RT-qPCR)

The RNA extracted from the preadipocytes of different transfection groups and transfection-induced precursor adipocytes was reverse-transcribed into cDNA. Briefly, using a reverse transcription kit (AG11701, AG, Changsha, China), 1 µg of total RNA was reverse-transcribed into cDNA. The stem-loop method was used to determine the expression level of miRNA. RT-qPCR was performed under special conditions (95 °C for 30 s, 40 cycles of 95 °C for 5 s and 60 °C for 30 s). Table 1 and Table 2 provide the starter sequences for mRNAs, miRNAs and circRNAs, respectively. Each experiment was carried out at least three times independently of each other. The housekeeping gene for miR-125a-3p is U6, whereas the housekeeping gene for circRNA-5335 and STAT3 is GAPDH. The expression levels of mRNA or miRNA were quantified by using the 2^−△△CT^ method and then normalized to GAPDH or U6 [27].

### 2.8. Western Blotting (WB)

The total proteins were extracted from the cells after cell lysis with RIPA (R0020, Solarbio, Beijing, China). A BCA Protein Assay Kit (PC0020, Solarbio, Beijing, China) was used to measure the protein concentration. The concentration-adjusted protein samples were resolved via SDS-PAGE (P0015L, Beyotime, Nanjing, China). The strips were transferred from the adhesive to a PVDF membrane (IPVH00010, Millipore, Bedford, MA, USA). The target protein content was detected using the luminescence imaging system (iBright FL 1000, Invitrogen, Carlsbad, CA, USA) [28], and the levels were compared with GAPDH (AC027, ABclonal, Wuhan, China). The used proteins were Color Mixed Protein Marker (11-245KD, PR1920, Solarbio, Beijing, China) and Prestained Protein Marker (MP102, Vazyme, Nanjing, China). The rabbit primary antibodies used were as follows: monoclonal antibody STAT3 (AF1492, Beyotime, Nanjing, China), polyclonal antibody P-STAT3 (bs1658R, Bioss, Beijing, China; AP0474, ABclonal, Wuhan, China) and PCNA (A0264, ABclonal, Wuhan, China). Horseradish peroxidase was conjugated to the goat anti-rabbit IgG antibody as the secondary antibody (ab97051, Abcam, Cambridge, UK).

### 2.9. Statistical Analysis 

In order to obtain mean ± SD data, these experiments were repeated three times. Data significance (*p* < 0.05) was analyzed via univariate ANOVA in SPSS 26.0 (Armonk, NY, USA). The data were plotted using GraphPad Prism 6 (San Diego, CA, USA), and the images were analyzed by Image J 1.8.0 (Bethesda, Rockville, MD, USA). 

## 3. Results

### 3.1. Expression Levels of circRNA-5335/miR-125a-3p/STAT3 during Sheep Preadipocyte Differentiation 

**Figure 1:**
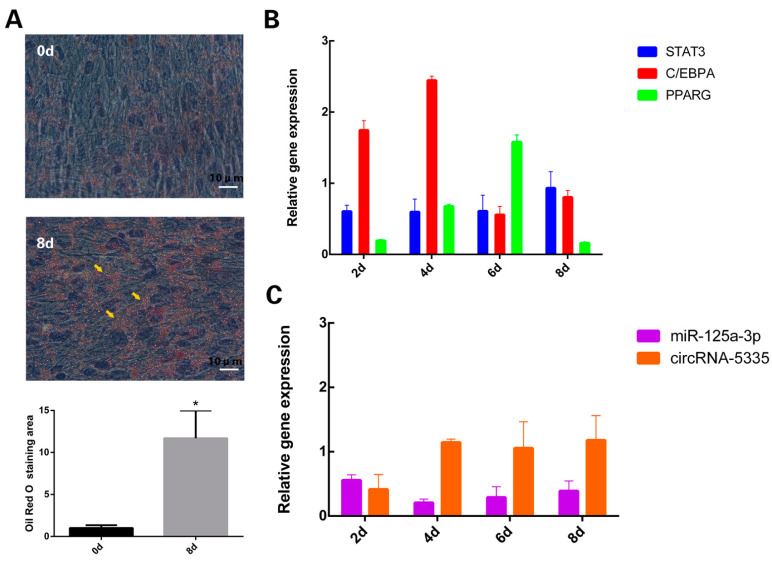
Changes in gene expression and lipid droplets during induced differentiation of preadipocytes: (**A**) Oil Red O staining of preadipocytes before and after differentiation. (**B**) Expression levels of target gene STAT3 and differentiation marker genes PPARG and C/EBPA during differentiation and maturation of adipocytes. (**C**) Change in expression of miR-125a-3p and circRNA-5335 during adipocyte differentiation. * *p* < 0.05. Bar: 10 μm. The yellow arrow points to the location of the grease droplet collection point.

The ORO staining and Image J analysis showed that the lipid droplets in mature adipocytes were significantly increased (Figure 1A). The expressions of STAT3, miR-125a-3p, circRNA-5335, PPARG and C/EBPA during sheep preadipocyte adipogenesis were examined. The results of the RT-qPCR analysis showed that the expression of the target gene STAT3 increased continuously during the differentiation of the preadipocytes. The differentiation marker gene C/EBPA was on the rise in the whole process. The STAT3 mRNA was similar to that observed for the differentiation marker gene C/EBPA. In addition, the differentiation marker gene PPARG increased during differentiation (Figure 1B). The expression of circRNA-5335 showed an upward trend during differentiation, while miR-125a-3p decreased in the first 4 days of sheep preadipocyte differentiation and then gradually increased (Figure 1C).

### 3.2. Knockdown of STAT3 Inhibits Differentiation Process of Preadipocytes

**Figure 2:**
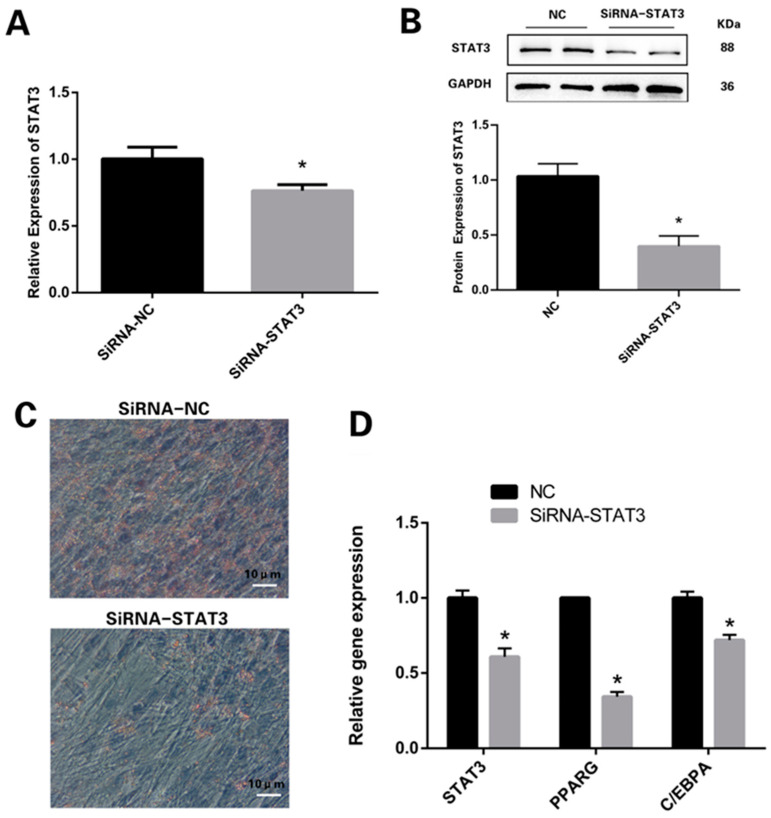
STAT3 regulated the differentiation of preadipocytes: (**A**) Functional verification of the transfection efficiency of SiRNA-STAT3. (**B**) STAT3 protein levels were detected by Western blotting and quantified by Image J. (**C**) Oil Red O staining identified a decrease in lipid droplets in cells after SiRNA-STAT3 interference. (**D**) Expression patterns of genes affected by SiRNA-STAT3 in differentiating sheep preadipocytes. * *p* < 0.05. Bar: 10 μm.

By constructing siRNA-STAT3 to knock down STAT3, the mRNA and protein expression levels in the knockdown group were obviously downregulated compared with those in the NC group (*n* = 3; Figure 2A,B; Figure S1). The Oil Red O staining of the differentiation revealed that STAT3 underexpression obviously impeded preadipocyte differentiation and fat formation in the sheep adipocytes (Figure 2C). The mRNA expression levels of STAT3, PPARG and C/EBPA were obviously reduced in the preadipocytes that underwent differentiation after transfection with siRNA-STAT3 (Figure 2D).

### 3.3. STAT3 Prevents Preadipocytes from Multiplying

**Figure 3:**
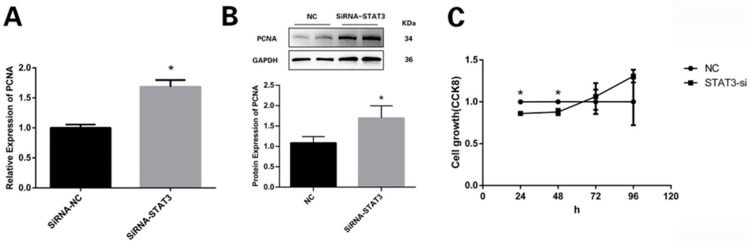
STAT3 regulated the proliferation of preadipocytes: (**A**) Relative abundances of proliferative marker gene PCNA. (**B**) The protein level of PCNA. (**C**) CCK8 assay comparing the cell growth rate in the SiRNA-STAT3 group with the NC group. * *p* < 0.05.

The mRNA and protein expression of the proliferation marker gene PCNA markedly increased in the SiRNA-STAT3 group (Figure 3A,B; Figure S1). The CCK8 detection showed that SiRNA-STAT3 obviously increased the proliferation rate of the preadipocytes compared to the NC group (Figure 3C). 

### 3.4. miR-125a-3p Regulates the Differentiation of Preadipocytes through STAT3 Signaling Pathway

**Figure 4:**
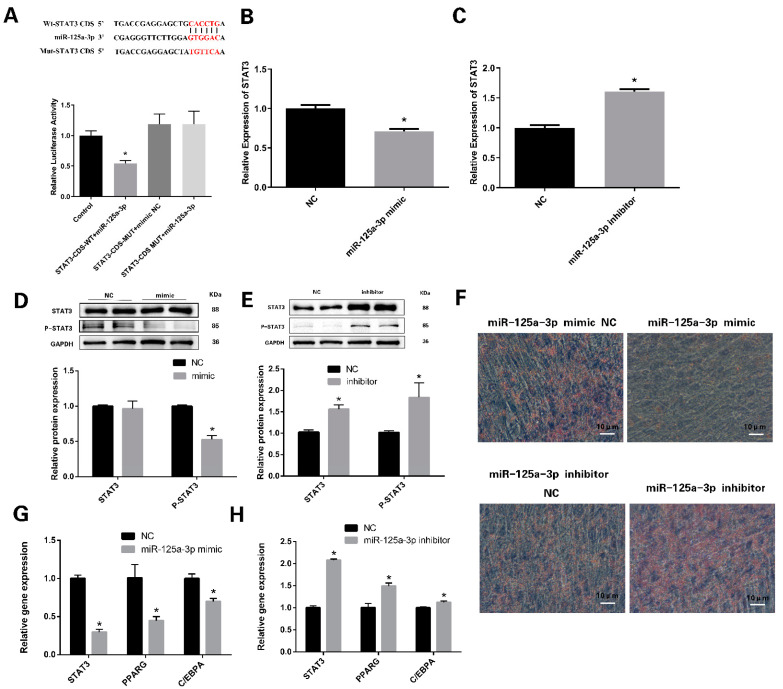
miR-125a-3p regulated preadipocyte differentiation by STAT3: (**A**) Dual-luciferase assay-detected target relationship between miR-125a-3p and STAT3. (**B**,**C**) Relative abundances of STAT3 determined using RT-qPCR analysis. (**D**,**E**) Relative abundances of STAT3 and P-STAT3 proteins identified from Western blotting analysis. (**F**) The lipid droplets in the adipocytes were stained with Oil Red O. (**G**) RT-qPCR analysis of STAT3, PPARG and C/EBPA after overexpressing miR-125a-3p in differentiating sheep preadipocytes. (**H**) The expression of target genes in differentiating sheep preadipocytes. * *p* < 0.05. Bar: 10 μm.

The results of the double luciferase experiment demonstrated that the luciferase activity in the WT-STAT3 (wild-type STAT3) group was significantly lower than other groups, indicating that the predicted targeting relationship between miR-125a-3p and STAT3 was valid (Figure 4A). The overexpression of miR-125a-3p markedly reduced the mRNA expression of STAT3 (Figure 4B), while miR-125a-3p inhibition increased the mRNA expression of STAT3 (Figure 4C). And the overexpression of miR-125a-3p (Figure S2) significantly lowered the levels of P-STAT3 (Figure 4D), while miR-125a-3p inhibition (Figure S3) increased the expression level of P-STAT3 (Figure 4E). 

To investigate the potential role of miR-125a-3p in adipogenesis, we transfected sheep preadipocytes with a miR-125a-3p mimic or miR-125a-3p inhibitor, respectively. After 24h of transfection, the mRNA expression levels of STAT3, PPARG and C/EBPA were significantly decreased in preadipocytes transfected with the miR-125a-3p mimic (Figure 4G), whereas the miR-125a-3p inhibitor markedly upregulated STAT3, PPARG and C/EBPA expression (Figure 4H). The Oil Red O staining of the differentiated cells showed that miR-125a-3p overexpression markedly inhibited preadipocyte differentiation and lipid accumulation in the sheep adipocytes. However, the inhibition of miR-125a-3p promoted lipid accumulation (Figure 4F). These results suggest that miR-125a-3p has a negative effect on sheep IMF adipogenesis.

### 3.5. miR-125a-3p Inhibits the Proliferation of Preadipocytes in Sheep

**Figure 5:**
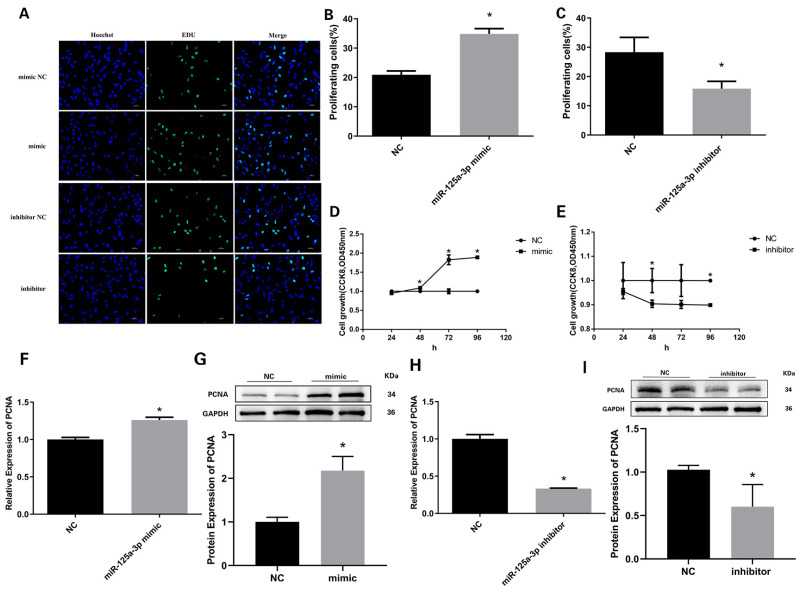
STAT3 and miR-125a-3p regulated preadipocyte proliferation: (**A**) The proliferation of preadipocytes was measured by EdU; the corresponding Image J analysis is shown in (**B**,**C**). (**D**) CCK8 results showed the proliferation of preadipocytes after increasing miR-125a-3p expression. (**E**) CCK8 experiment exhibited the cell proliferation rate in the miR-125a-3p inhibitor group. (**F**) Expression of PCNA after overexpression of miR-125a-3p. (**G**) Relative abundances of PCNA com-pared with the NC group in preadipocytes after treatment with mimics. (**H**) RT-qPCR analysis of the expression of proliferation marker gene PCNA after miR-125a-3p was inhibited. (**I**) Western blotting was used to detect both PCNA and GAPDH levels in the NC group and the inhibition group. * *p* < 0.05. Bar: 20μm.

After transfecting miR-125a-3p mimics or miR-125a-3p inhibitors, the EdU cell proliferation assay showed that the overexpression of miR-125a-3p significantly increased the number of EdU-positive preadipocytes, while the inhibition of miR-125a-3p significantly decreased the number of EdU-positive preadipocytes (Figure 5A,C). Similarly, CCK8 detection confirmed that miR-125a-3p mimics significantly increased the total amount of proliferating preadipocytes compared with the negative control group (Figure 5D). In contrast, the total number of cells transfected with miR-125a-3p inhibitors was markedly reduced compared with the inhibitor-negative control group (Figure 5E). In summary, these results reveal that miR-125a-3p can promote the proliferation of sheep preadipocytes. The mRNA and protein expression of PCNA was markedly increased in the miR-125a-3p mimic group (Figure 5F,G; Figure S2), while the miR-125a-3p inhibitor (Figure S3) significantly downregulated PCNA expression (Figure 5H,I). Collectively, these results indicate that miR-125a-3p can promote the proliferation of sheep preadipocytes, while STAT3 inhibits proliferation.

### 3.6. CircRNA-5335 Regulates the Expression of STAT3 by Binding to miR-125a-3p

**Figure 6:**
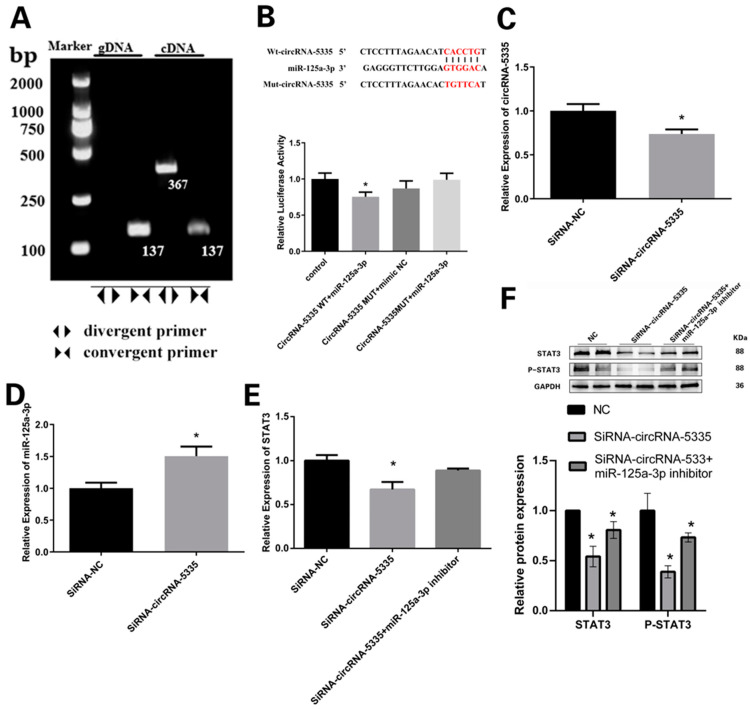
circRNA-5335 has a strong targeting relationship with miR-125a-3p: (**A**) The absence of products in the first lane indicates that divergent primers were unable to amplify products in gDNA, whereas the third lane shows the amplification from cDNA. Convergent primers amplified products from both gDNA and cDNA. (**B**) Target relationship between circRNA-5335 and miR-125a-3p. (**C**) SiRNA-circRNA-5335 interfered with circRNA-5335 expression. (**D**) Relative abundances of miR-125a-3p after inhibiting circRNA-5335. (**E**) The expression of STAT3 was affected by SiRNA-circRNA-5335, and eliminated by miR-125a-3p inhibitors. (**F**) Western blotting analysis of SiRNA-circRNA-5335 and miR-125a-3p inhibitors affected by the protein levels of STAT3 and P-STAT3, and eliminated by miR-125a-3p inhibitors. * *p* < 0.05.

Both of the designed convergent and divergent primers produced the expected targets. The convergent primers were amplified products from both gDNA and cDNA, while the divergent primers were only amplified products from the cDNA. These results verified the existence of circRNA-5335 in both gDNA and cDNA, indicating that circRNA-5335 forms a circular structure in the preadipocytes. However, its circular form was only detected in the total RNA (Figure 6A). The dual-luciferase assay showed a significant difference in the luciferase activity between the WT-circRNA-5335 group and the other groups (Figure 6B), indicating that the previously predicted targeting relationship between circRNA-5335 and miR-125a-3p was established.

The expression level of circRNA-5335 was markedly downregulated (Figure 6C) and the expression of miR-125a-3p was increased (Figure 6D) by SiRNA-circRNA-5335 compared with those in the NC group. Similarly, the expression level of STAT3 was significantly inhibited by SiRNA-circRNA-5335. Meanwhile, the interference caused by circRNA-5335 could be remedied by the miR-125a-3p inhibitor (Figure 6E,F; Figure S4). 

### 3.7. circRNA-5335/miR-125a-3p/STAT3 Regulates the Differentiation and Proliferation of Preadipocytes

**Figure 7:**
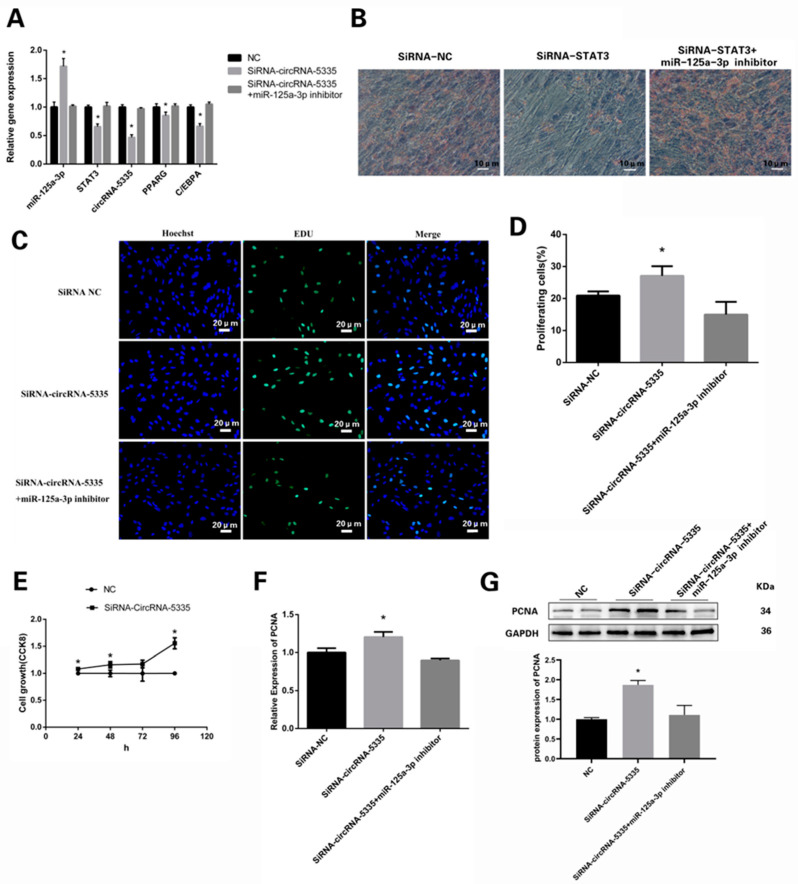
Effects of SiRNA-circRNA-5335 on the differentiation and proliferation of preadipocytes: (**A**) Relative abundances of target genes after inhibiting circRNA-5335 in differentiating sheep preadipocytes. (**B**) Oil Red O analysis of the target gene expression after 8 days of differentiation. Bar: 10 μm. (**C**) EdU staining showed the proliferation of preadipocytes after inhibiting circRNA-5335; the corresponding image-J analysis is shown in (**D**) Bar: 20 μm. (**E**) The cell proliferation rate of the SiRNA-circRNA-5335 group was tested by the CCK8 method. (**F**) Relative abundances of PCNA detected by RT-qPCR. (**G**) Analysis of the relative abundances of PCNA protein. * *p* < 0.05.

The mRNA levels of STAT3 and differentiation marker genes including PPARG and C/EBPA were downregulated in the SiRNA-circRNA-5335-transfected adipocytes, while these changes were reversed by the miR-125a-3p inhibitor (Figure 7A). Oil Red O staining showed that downregulated circRNA-5335 markedly inhibited the preadipocyte differentiation and lipid accumulation in sheep cell culture (Figure 7B).

We also used EdU and CCK8 assays to verify whether circRNA-5335 functions in targeting sheep preadipocyte proliferation. After the SiRNA-circRNA-5335 was transfected, the EdU cell proliferation assay demonstrated that the inhibition of circRNA-5335 significantly increased the amount of EdU-positive preadipocytes, while the diversity caused by SiRNA-circRNA-5335 could be reversed by the overexpression miR-125a-3p (Figure 7C,D). Invariably, CCK8 detection showed that SiRNA-circRNA-5335 significantly increased the quantity of preadipocyte proliferation compared with the negative control group (Figure 7E). In addition, the mRNA and protein levels of the proliferation marker gene PCNA were upregulated in the SiRNA-circRNA-5335-transfected preadipocytes (Figure 7F,G; Figure S4). Conversely, these changes could be reversed by the overexpression of miR-125a-3p. Collectively, these results demonstrate that circRNA-5335 can inhibit the proliferation of sheep preadipocytes, but circRNA-5335 has a positive effect on sheep adipogenesis.

## 4. Discussion

Understanding the molecular regulation of IMF accumulation in sheep is of great significance for improving mutton quality and production efficiency [29]. In this experiment, we examined the role of the circRNA-5335/miR-125a-3p/STAT3 pathway in adipogenesis. 

We studied the effect of STAT3 on the preadipocyte differentiation and subsequent adipogenesis in sheep. Previous studies have reported some pathways of STAT3 involved in adipogenesis, such as the JAK2/STAT3 pathway, which regulates C/EBP-β transcription in the early stages of preadipocyte differentiation [4]. The expression of STAT3 was found to increase during adipogenesis and differentiation in 3T3-L1 cells [12], and DMF targets STAT3 to inhibit adipocyte differentiation [30,31]. Consistent with our results, the expression of STAT3 increased during the induction of differentiation; meanwhile, we confirmed that STAT3 promotes the differentiation of preadipocytes in sheep.

The significant genetic influence of STAT3 on both growth and development in sheep suggests that STAT3 can be used as a marker gene for the selection of growth traits in sheep [32]. A study showed that an inhibitor of JAK2/STAT3 suppressed the growth of preadipocytes [33]. Similarly, STAT3 knockdown using siRNA-STAT3 blocked the cell cycle progression of both preadipocytes and early differentiating cells [34]. Likewise, we found that in the WAT tissue of sheep, inhibiting STAT3 expression promoted preadipocyte proliferation.

Moreover, some studies have shown that highly downregulated miRNAs may promote lipogenesis by activating target genes including those associated with fatty acid metabolism and fat formation and differentiation. For instance, miR-138-5p regulates preadipocyte differentiation by modulating EZH2 expression [35]. Similarly, adipogenesis-related miR-143-3p can regulate lipid deposition through its downstream target gene IGF2R [36]. Similar to the study above, our experiments indicate that miR-125a-3p targets STAT3 to decrease the lipid droplets in preadipocytes during adipogenesis. These findings indicate that miR-125a-3p may play a role as a negative regulator for preadipocyte differentiation.

In addition, previous analysis confirms that ssc-miR-149 and ssc-miR-425-3p participate in preadipocyte proliferation in pigs [37], suggesting that microRNA may play an important regulatory role in adipogenesis. We found that miR-125a-3p could slow down the rate of cell proliferation in a series of experiments. These results indicate that interfering with miR-125a-3p affects the expression of its target gene STAT3, which in turn affects the differentiation and proliferation of preadipocytes in sheep.

CircRNAs are a class of non-coding RNAs that form covalently closed loops and have diverse biological functions. CircRNAs can interact with microRNAs or act as miRNA sponges [38,39], thereby affecting the expression quantity of target genes. CircRNAs also play transcriptional regulatory roles in adipocytes, influencing lipid biosynthesis and metabolism. In recent years, circRNAs have attracted increasing attention in the field of animal science, especially in the lipid metabolism and regulation of adipogenesis. We previously screened for circRNAs with significant differences between 2-month-old and 12-month-old sheep, and identified circRNA-5335/miR-125a-3p/STAT3 as a candidate [20]. We found that the expression of circRNA-5335 increased continuously during the induction of differentiation, suggesting that it may be involved in the adipogenic process. Compared with gDNA, we observed that only the cDNA obtained from the RNase R-treated total RNA showed a specific band corresponding to the backsplice junction fragment [40], which verified that circRNA-5335 exists in preadipocytes as a circular molecule.

Several studies have explored the circRNA/microRNA/gene axis to open up more possibilities for gene regulatory networks. Several recent findings revealed the role of circRNAs in fat formation, insulin resistance, obesity, obesity-induced inflammation and the browning of white fat [41]. The expression of circPPRA positively affects IMF content [42], and CircArhgap5-2 is conserved in human adipocytes, indicating that circRNAs have a crucial regulatory role in preadipocyte differentiation and metabolism [43]. Thus, we investigated the role of circRNA-5335 in the differentiation of sheep preadipocytes and its potential mechanism of regulating intramuscular fat (IMF) deposition. Our experimental results showed that circRNA-5335 regulates the differentiation of preadipocytes and plays a role in adipogenesis. We verified that circRNA-5335 increased the expression of STAT3 and differentiation marker genes and the number of fat drops, thus promoting preadipocyte differentiation. Therefore, we identified circRNA-5335 as a positive regulator of preadipocyte differentiation that has an opposite function to miR-125a-3p and can be interfered with by miR-125a-3p inhibitors.

CircRNA-Circ-ATXN2 is a new type of age-related circRNA that can inhibit proliferation and promote cell lipogenesis and death in adipose-tissue-derived stromal cells [44]. CircFUT10 binds to let-7c and targets bovine adipocyte PPARGC1B to promote cell proliferation and inhibit cell differentiation [1]. The expression level of the proliferation marker gene PCNA and the number of new cells could be inhibited by circRNA-5335, thus inhibiting preadipocyte proliferation. In addition, we co-transfected sheep preadipocytes with SiRNA-circRNA-5335 and a miR-125a-3p inhibitor and confirmed that circRNA-5335 acts as a sponge of miR-125a-3p to regulate IMF development. This means that miR-125a-3p may bind to circRNA-5335 and regulate the proliferation of preadipocytes. 

## 5. Conclusions

**Figure 8:**
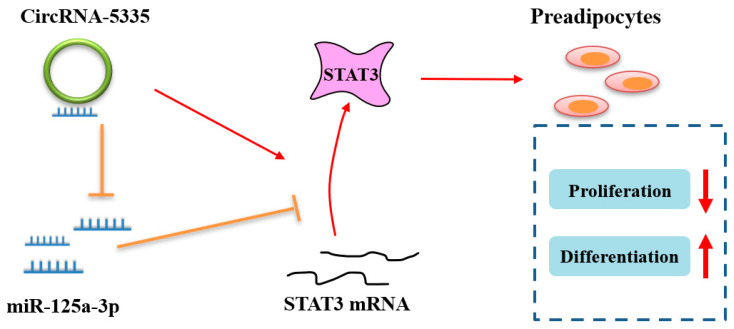
Schematic illustration of the circRNA-5335/miRNA-125a-3p/STAT3 axis. CircRNA-5335 plays its role by interacting with STAT3 mRNA and competing with miRNA-125a-3p, thereby reducing its inhibition of proliferation and promoting differentiation.

In summary, our study verified that circRNA-5335, as a novel sponge, can regulate the combination of miR-125a-3p and STAT3 (Figure 8), thereby affecting the differentiation and proliferation of sheep preadipocytes. This is of great significance for IMF formation and a field of great value to explore. On the one hand, it offers a new avenue of research to investigate the regulatory mechanism of preadipocyte proliferation and differentiation in sheep. On the other hand, the newly discovered regulatory genes can be utilized as molecular markers to identify sheep with high-quality meat. Genetic engineering technology can be employed to enhance the meat quality of sheep.

## Figures and Tables

**Table 1:** Information on genes for qPCR.

Target Gene	Primer Sequence (5′-3′)	Product Size (bp)	Accession Number
*STAT3*	F:5′-GGAACCTTACACCAAACAGCA-3′ R:5′-AGGGTAGAGATAGACCAGCGG-3′	115	XM_042255967.1
*PCNA*	F:5′-GTGGAGAACTTGGAAATGGAA-3′ R:5′-GAGACAGTGGAGTGGCTTATG-3′	153	XM_004014340.5
*PPARG*	F:5′-CATTTCTGCTCCGCACTAC-3′ R:5′-TGGAACCCTGACGCTTT-3′	234	NM_001100921.1
*CEBPA*	F:5′-GTGGAGACGCAACAGAAGGT-3′ R:5′-AGTTCGCGGCTCAGTTGTT-3′	83	NM_001308574.1
*GAPDH*	F:5′-AAGTTCAACGGCACAGTCA-3′ R:5′-ACCACATACTCAGCACCAGC-3′	125	NM_001190390.1

**Table 2:** Information on miRNA and circRNA for qPCR.

Target Gene	Primer Sequence (5′-3′)
CircRNA-5335 convergent	F: CGACAAAGAGGAAATAGCAAT R: ACAGGTGATGTTCTAAAGGAG
CircRNA-5335 divergent	F: CACACTCTTGGATTCAGCAGC R: CCTCTTTGATAGGACACTCGT
miRNA-125a-3p	F: CGCGACAGGTGAGGTTCTT R: AGTGCAGGGTCCGAGGTATT RT:GTCGTATCCAGTGCAGGGTCCGAGGTATTCGCACTGGATACGACGCTCCC
U6	F: GGAACGATACAGAGAAGATTAGC R: TGGAACGCTTCACGAATTTGCG

## Data Availability

The corresponding author can provide the datasets used and/or analyzed during the current study upon reasonable request.

## Supplementary Materials

The following supporting information can be downloaded at https://www.mdpi.com/article/10.3390/vetsci11020070/s1: The RN-Seq data were submitted to the SRA database under accession number SRR12247890. Figure S1: Alterations in the expression of GAPDH, STAT3, P-STAT3 and PCNA proteins were observed in preadipocytes treated with either a miR-125a-3p inhibitor or a negative control (NC) construct. Figure S2: Alterations in the expression of GAPDH, STAT3, P-STAT3 and PCNA proteins were observed in preadipocytes treated with either a miR-125a-3p mimic or a negative control (NC) construct. Figure S3: Alterations in the expression of GAPDH, STAT3, P-STAT3 and PCNA proteins were observed in preadipocytes treated with SiRNA-circRNA-5335 and co-transfected with miR-125a-3p or a negative control (NC) construct. Figure S4. Alterations in the expression of GAPDH, STAT3, P-STAT3 and PCNA proteins were observed in preadipocytes treated with either a SiRNA-STAT3 or a negative control (NC) construct.
