# Supplementary material for: CircRNA-5335 Regulates the Differentiation and Proliferation of Sheep Preadipocyte via the miR-125a-3p/STAT3 Pathway
Source: Vet Sci. 2024 Feb 4;11(2):70. doi: 10.3390/vetsci11020070 (PMC10891738; doi:10.3390/vetsci11020070)
Supplement: Supplementary file 1 [file vetsci-11-00070-s001.zip › vetsci-2770365-supplementary.pdf]

Supplementary Materials: CircRNA-5335 Regulates the Differentiation and Proliferation of Sheep Preadipocyte via the miR-125a-3p/STAT3 Pathway

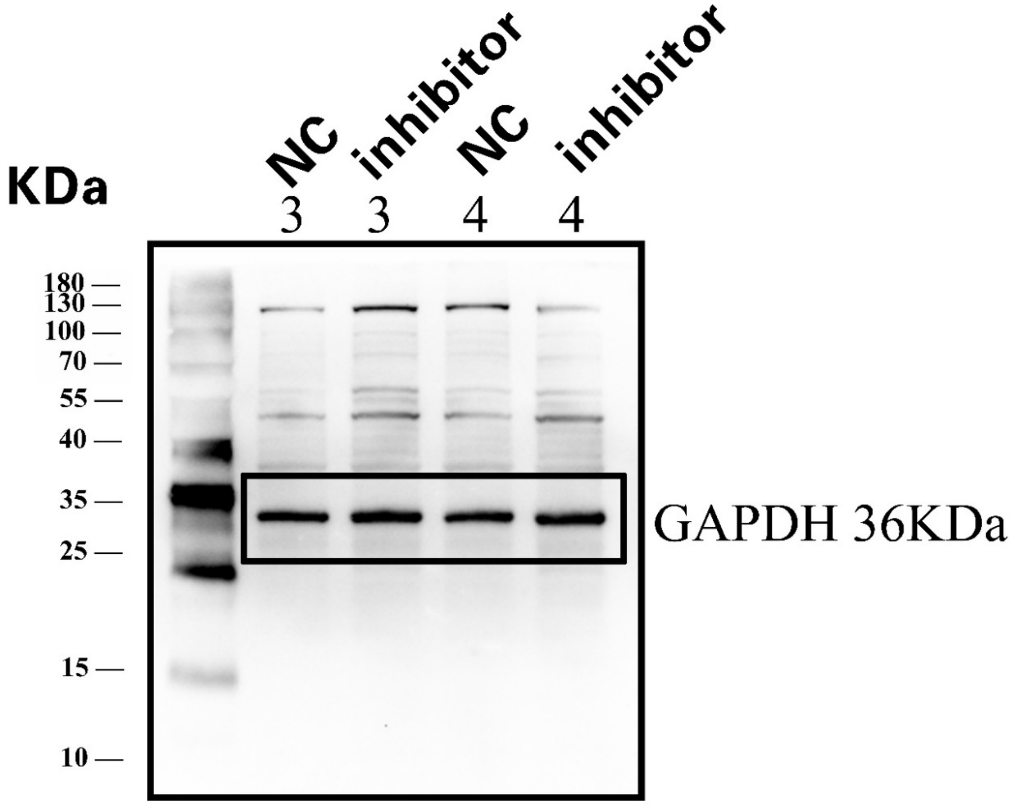
KDa
NC 3
inhibitor 3
NC 4
inhibitor 4
180
130
100
70
55
40
35
25
15
10
GAPDH 36KDa

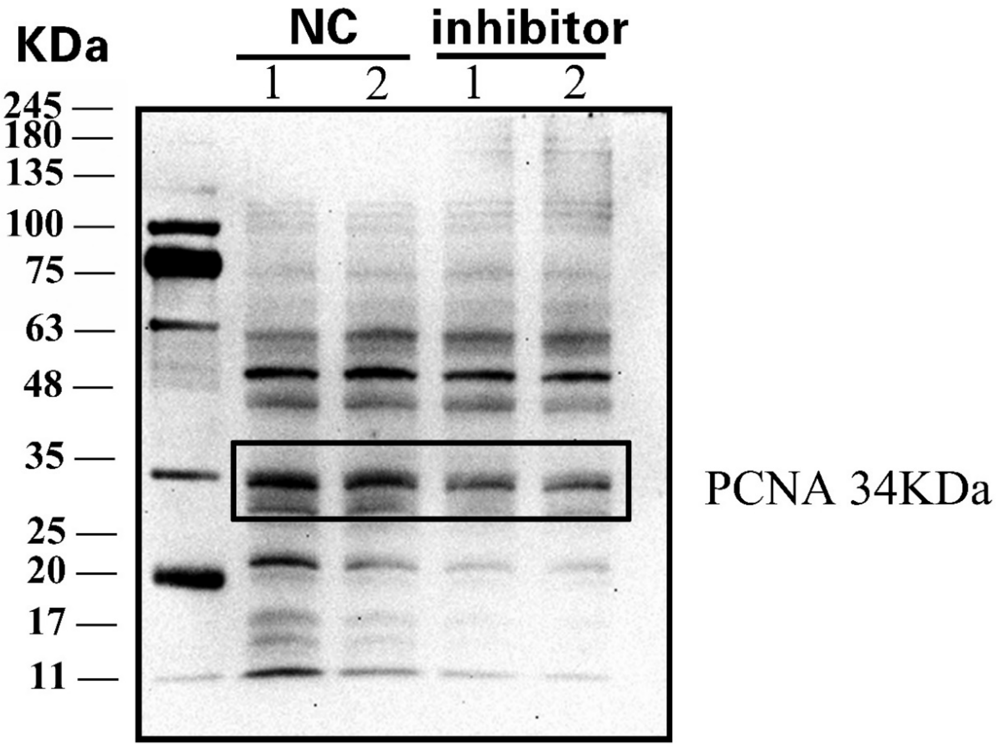
KDa
NC
inhibitor
1
2
1
2
245
180
135
100
75
63
48
35
25
20
17
11
PCNA 34KDa

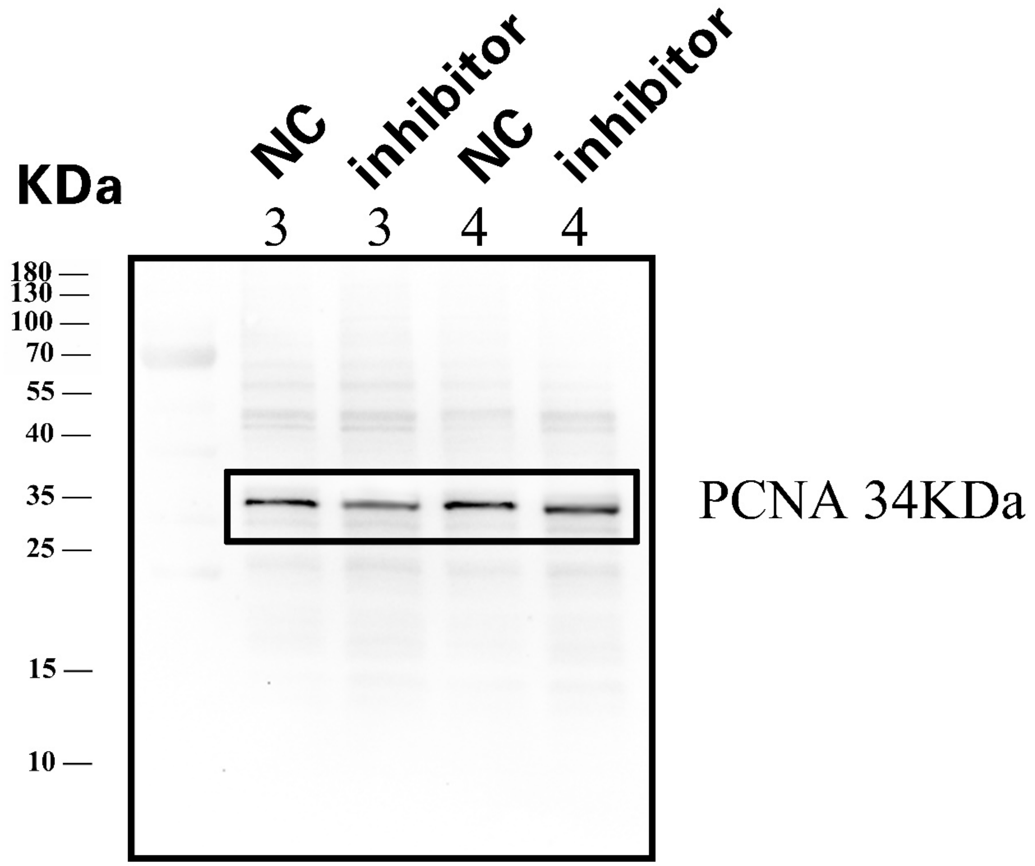

KDa
NC
inhibitor
NC
inhibitor
3
3
4
4
180
130
100
70
55
40
35
25
15
10
PCNA 34KDa

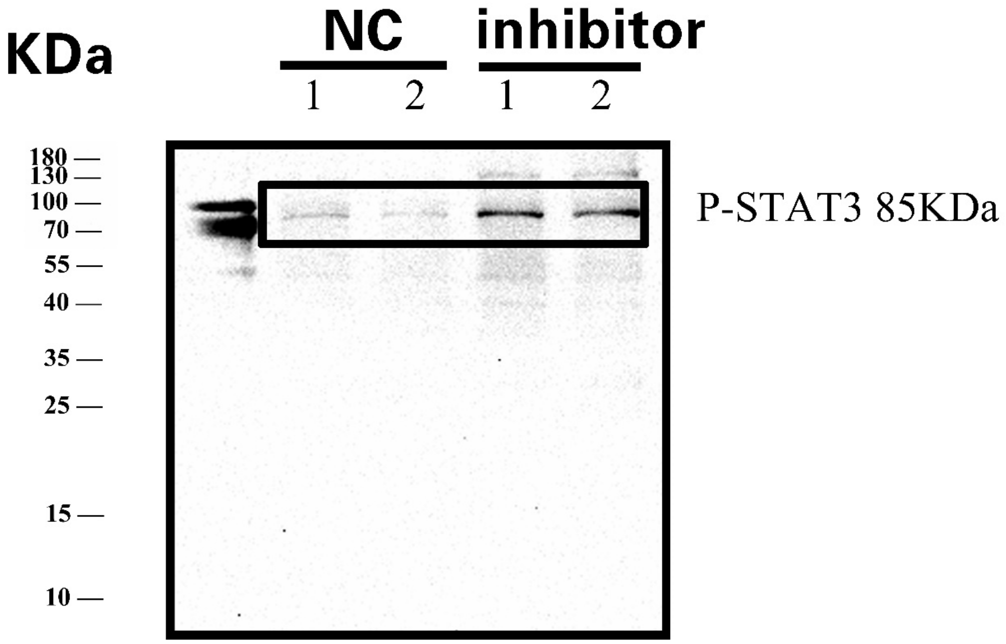

KDa
NC
inhibitor
1
2
1
2
180
130
100
70
55
40
35
25
15
10
P-STAT3 85KDa

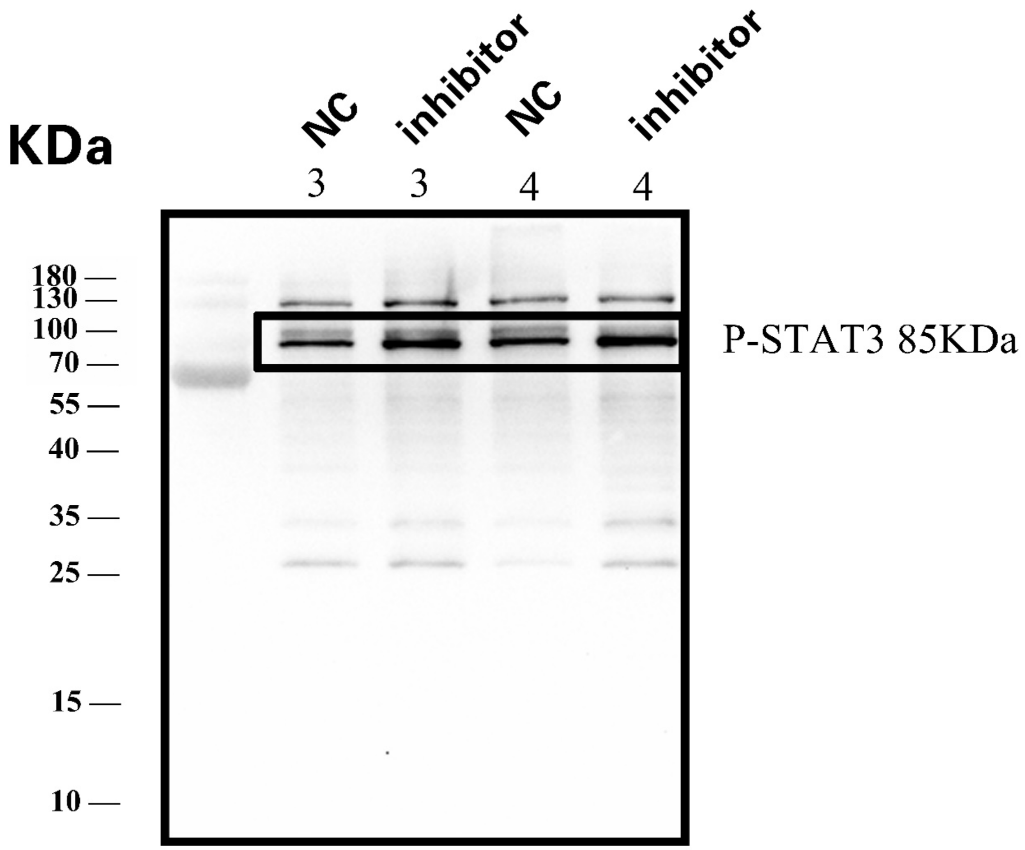
KDa
NC
inhibitor
NC
inhibitor
3
3
4
4
180
130
100
70
55
40
35
25
15
10
P-STAT3 85KDa

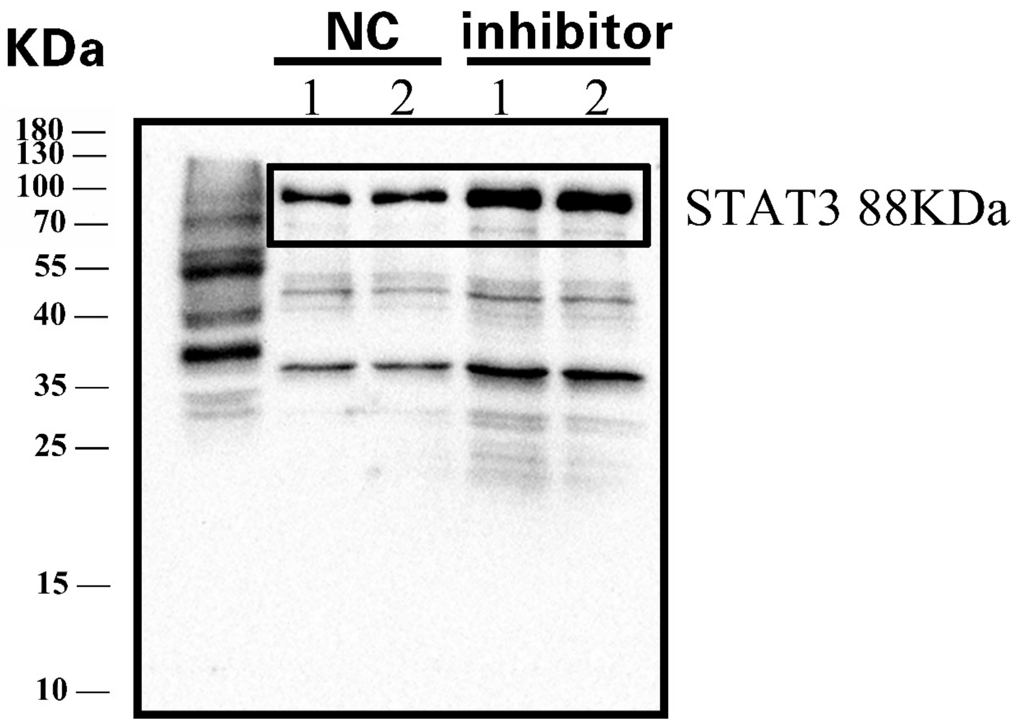
KDa
NC
inhibitor
1
2
1
2
180
130
100
70
55
40
35
25
15
10
STAT3 88KDa

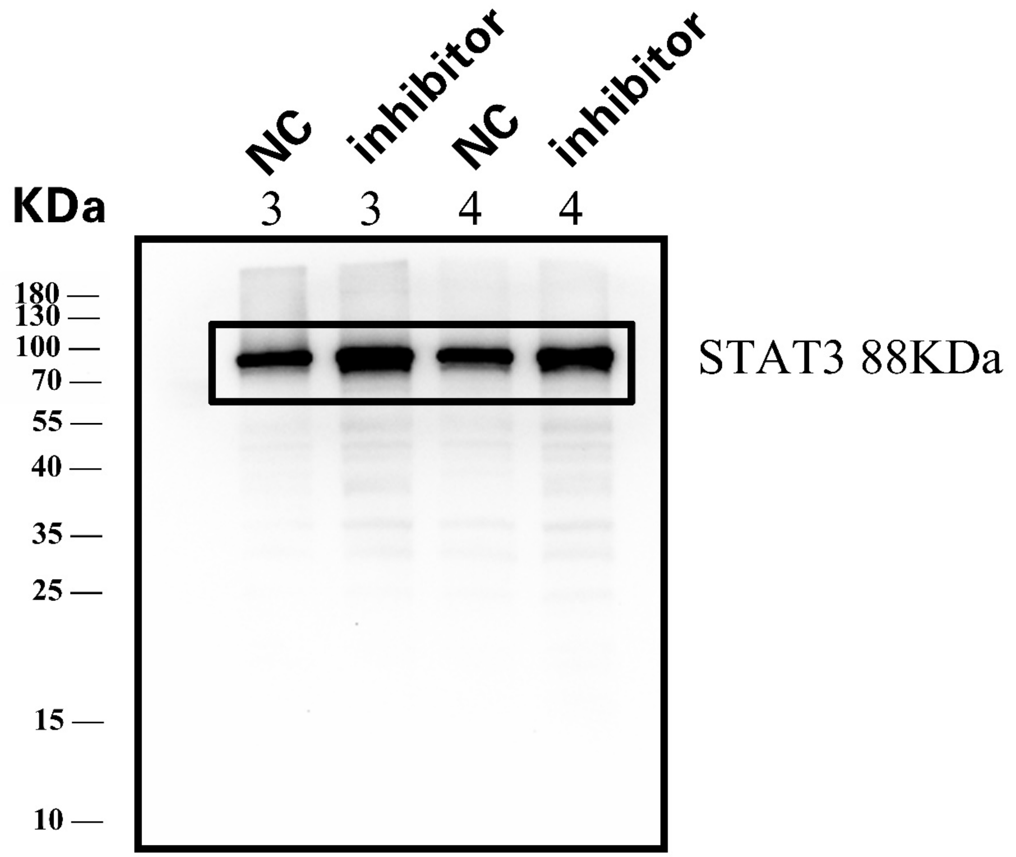

Figure S1. Alterations in the expression of GAPDH, STAT3, P-STAT3, and PCNA proteins were observed in preadipocytes treated with either a miR-125a-3p inhibitor or a negative control (NC) construct.

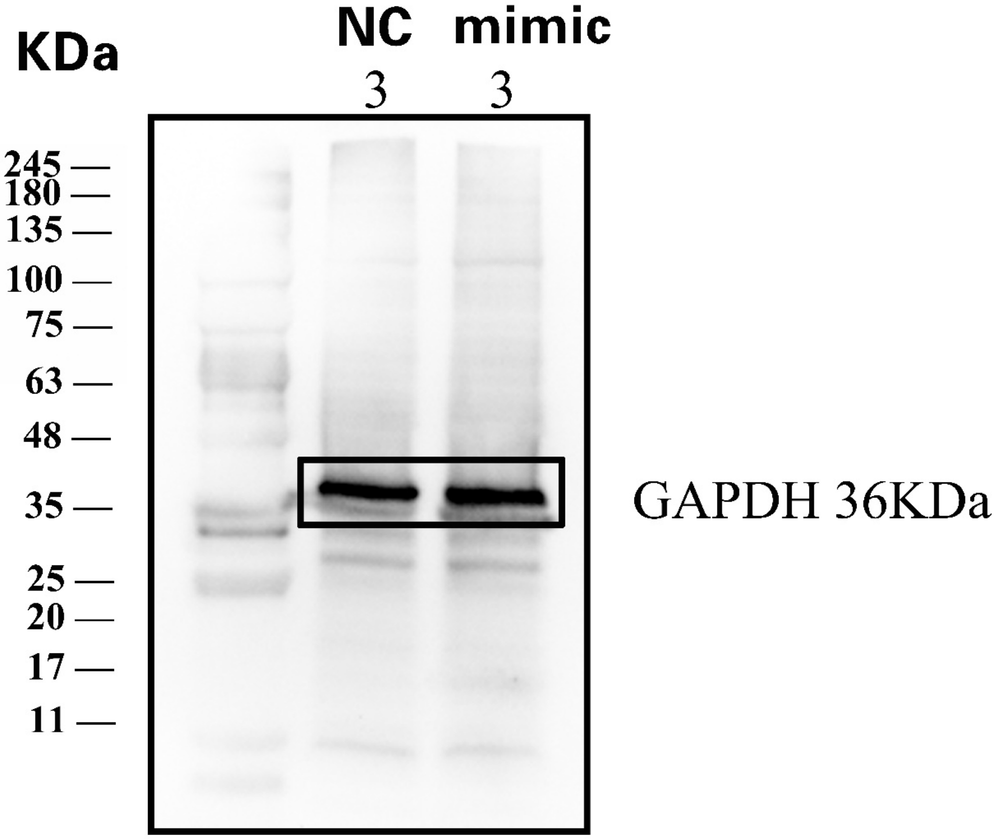
KDa
NC
mimic
3
3
245
180
135
100
75
63
48
35
25
20
17
11
GAPDH 36KDa

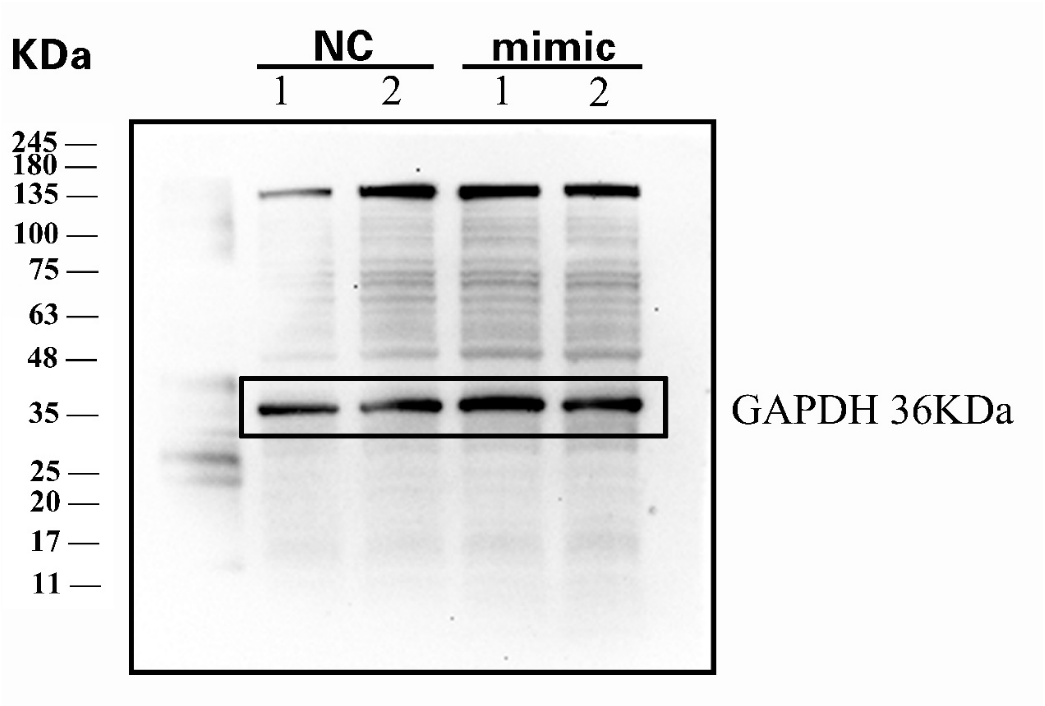
KDa
NC
mimic
1
2
1
2
245
180
135
100
75
63
48
35
25
20
17
11
GAPDH 36KDa

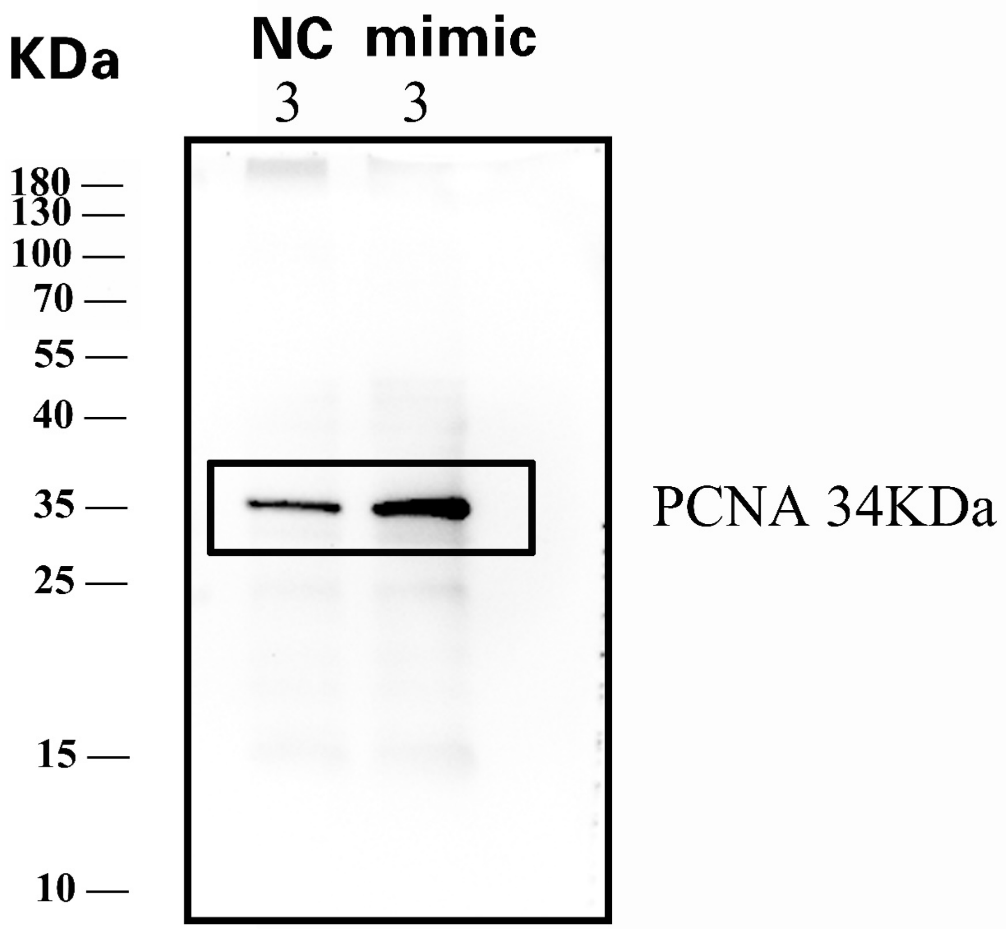

KDa
NC mimic
3 3
180
130
100
70
55
40
35
25
15
10
PCNA 34KDa

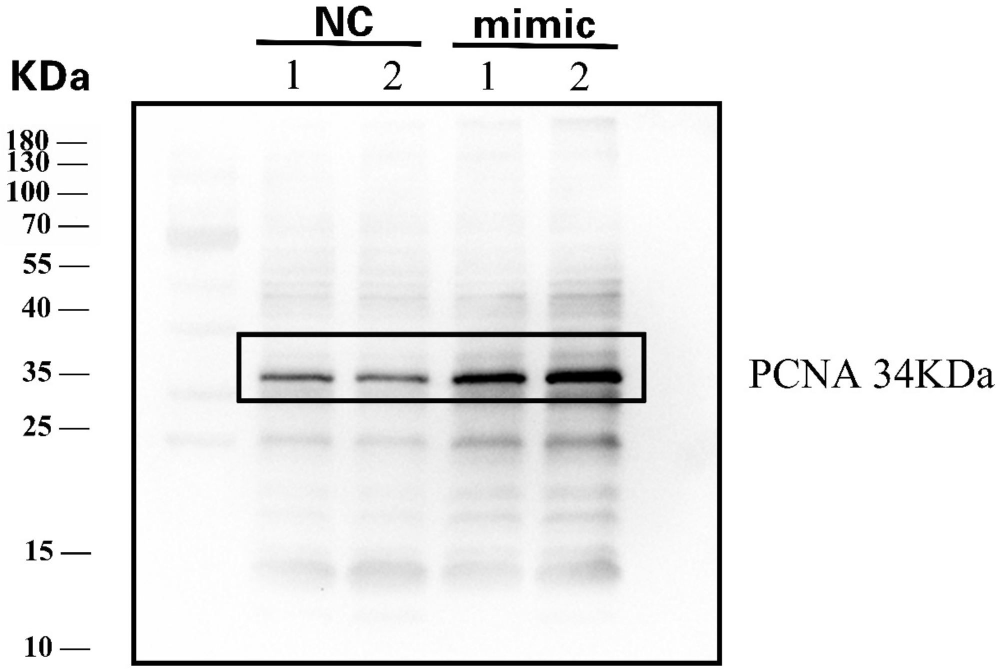

KDa
NC
mimic
1 2 1 2
180
130
100
70
55
40
35
25
15
10
PCNA 34KDa

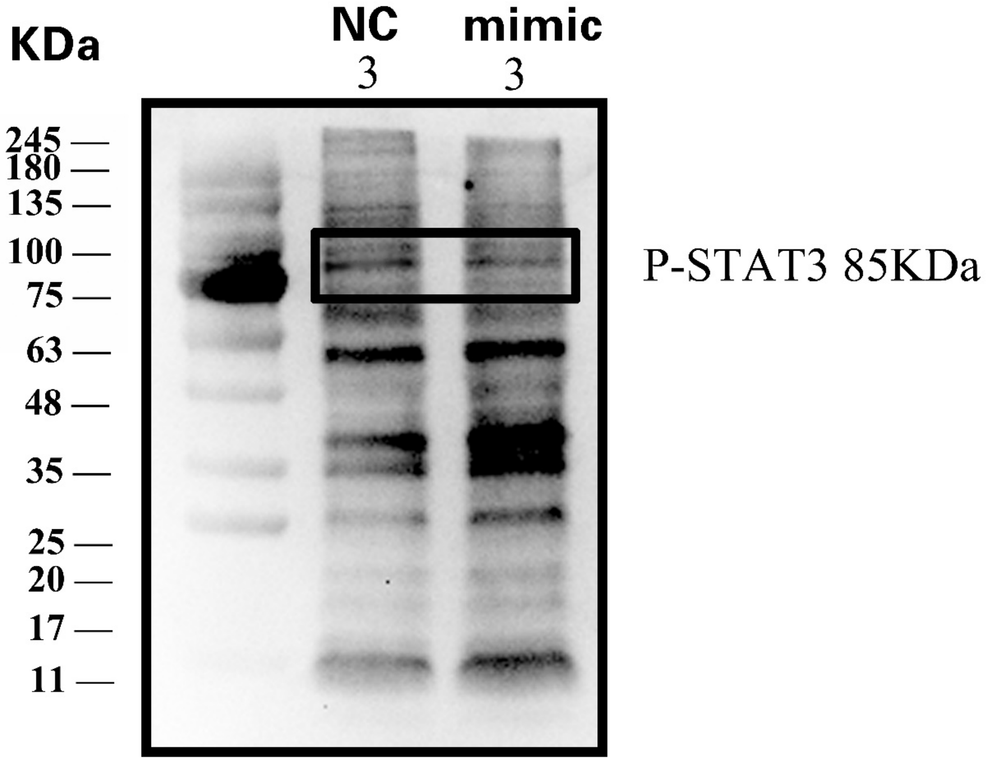
KDa
NC
3
mimic
3
245
180
135
100
75
63
48
35
25
20
17
11
P-STAT3 85KDa

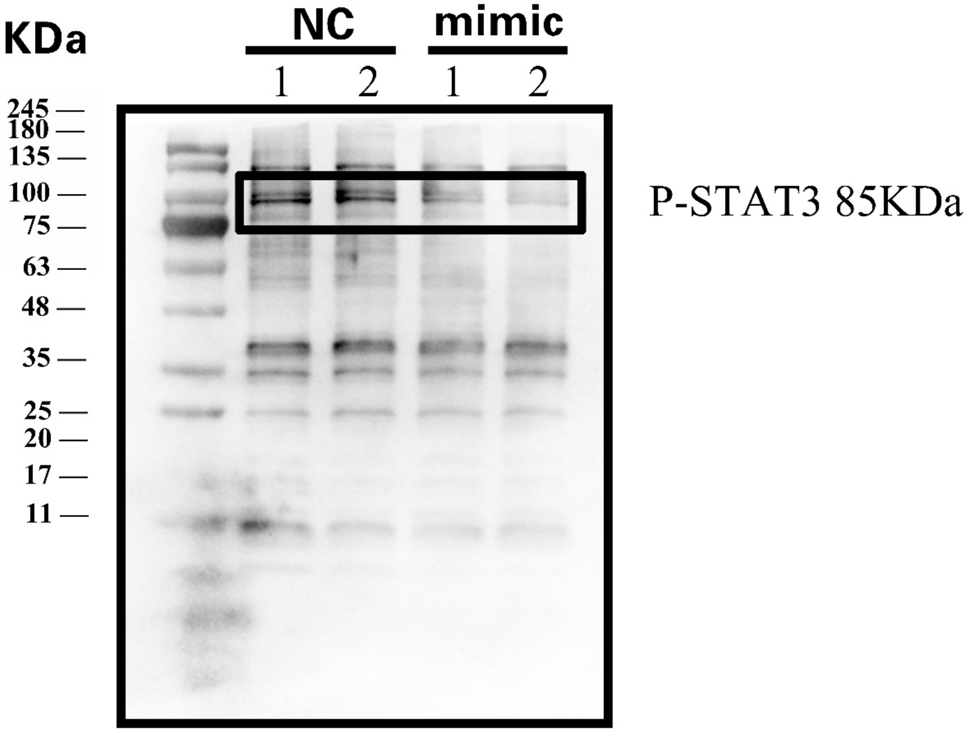
KDa
NC
mimic
1
2
1
2
245
180
135
100
75
63
48
35
25
20
17
11
P-STAT3 85KDa

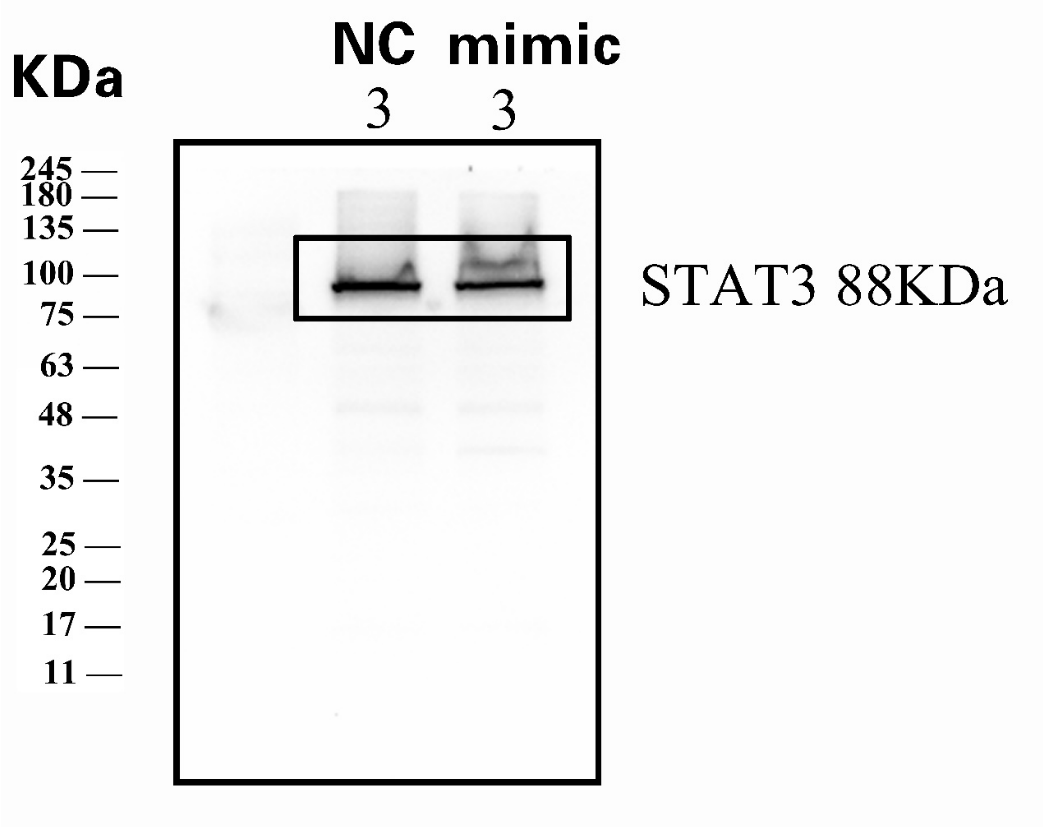

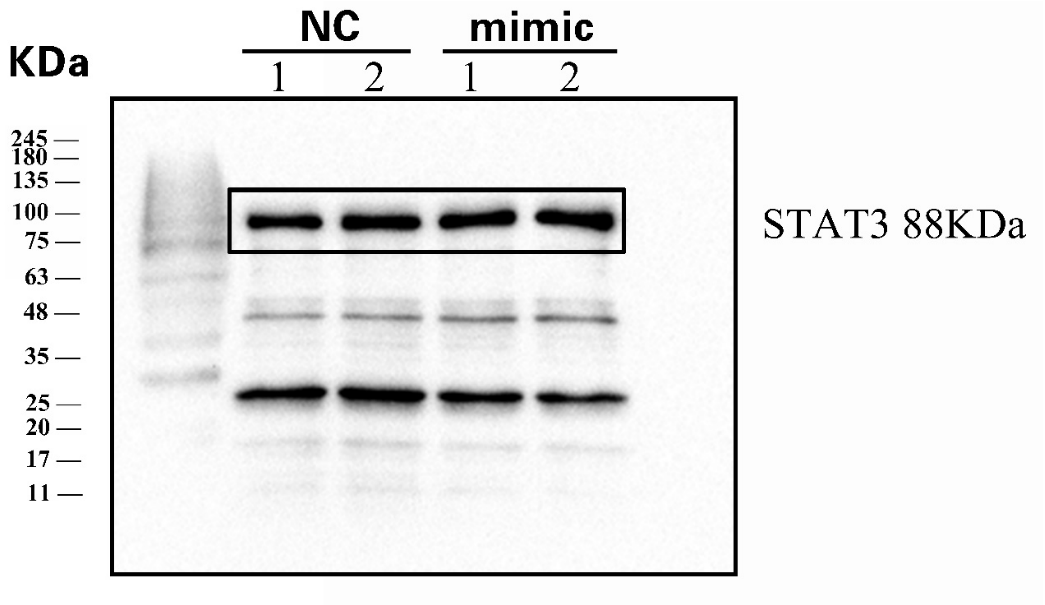

Figure S2. Alterations in the expression of GAPDH, STAT3, P-STAT3, and PCNA proteins were observed in preadipocytes treated with either a miR-125a-3p mimic or a negative control (NC) construct.

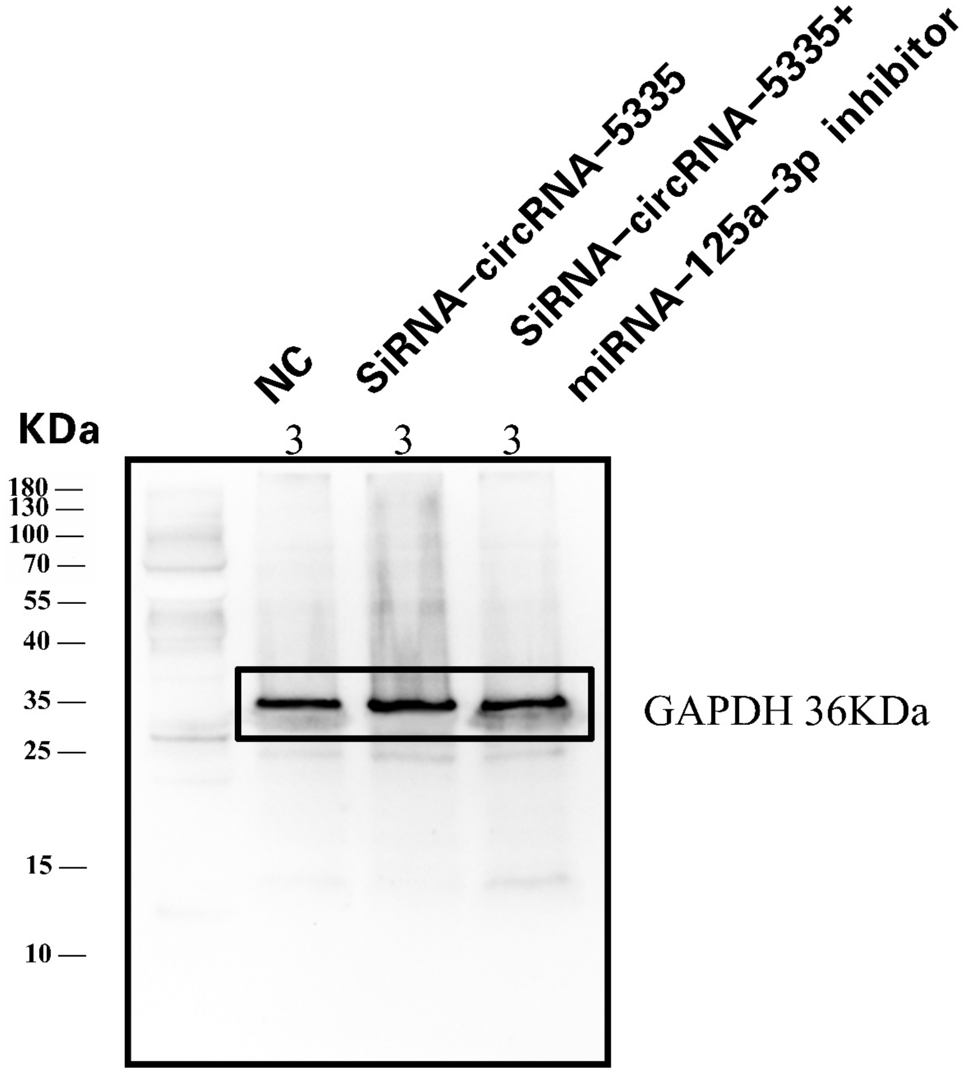

NC
SiRNA-circRNA-5335
SiRNA-circRNA-5335+
miRNA-125a-3p inhibitor
KDa
3
3
3
180
130
100
70
55
40
35
25
15
10
GAPDH 36KDa

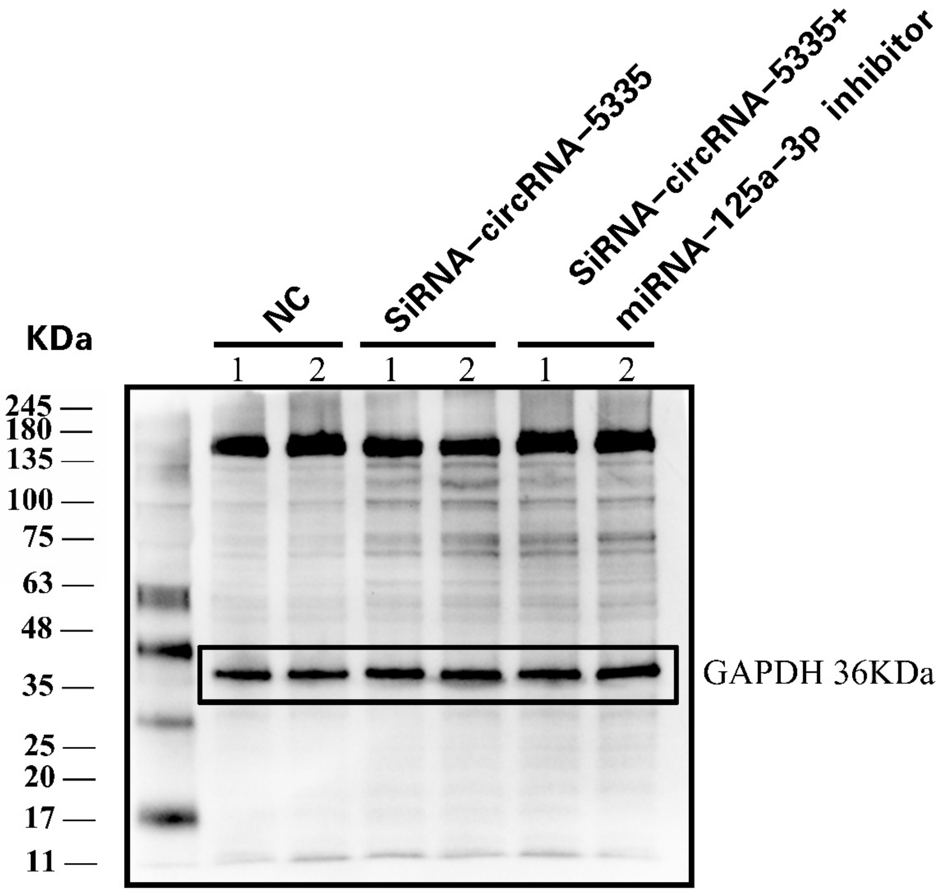

KDa
NC
SiRNA-circRNA-5335
SiRNA-circRNA-5335+
miRNA-125a-3p inhibitor
1
2
1
2
1
2
245
180
135
100
75
63
48
35
25
20
17
11
GAPDH 36KDa

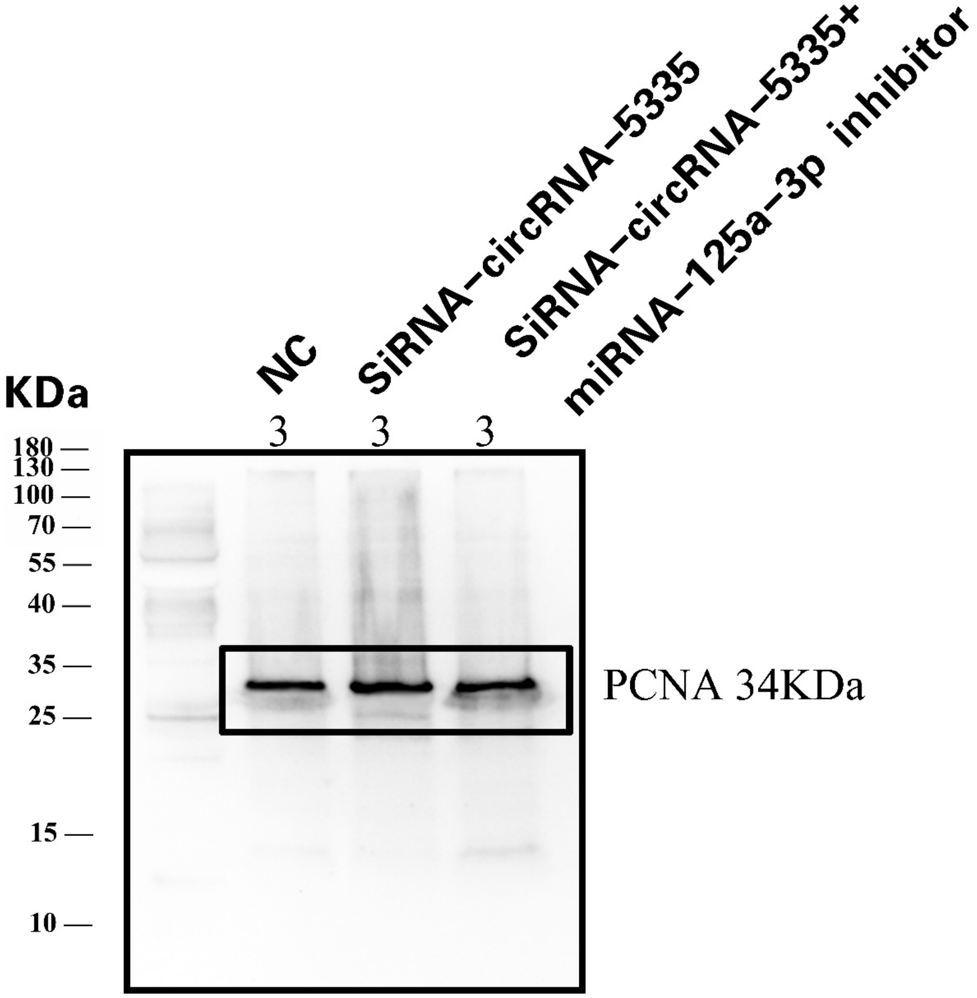
KDa
NC
SiRNA-circRNA-5335
SiRNA-circRNA-5335+
miRNA-125a-3p inhibitor
3
3
3
180
130
100
70
55
40
35
25
15
10
PCNA 34KDa

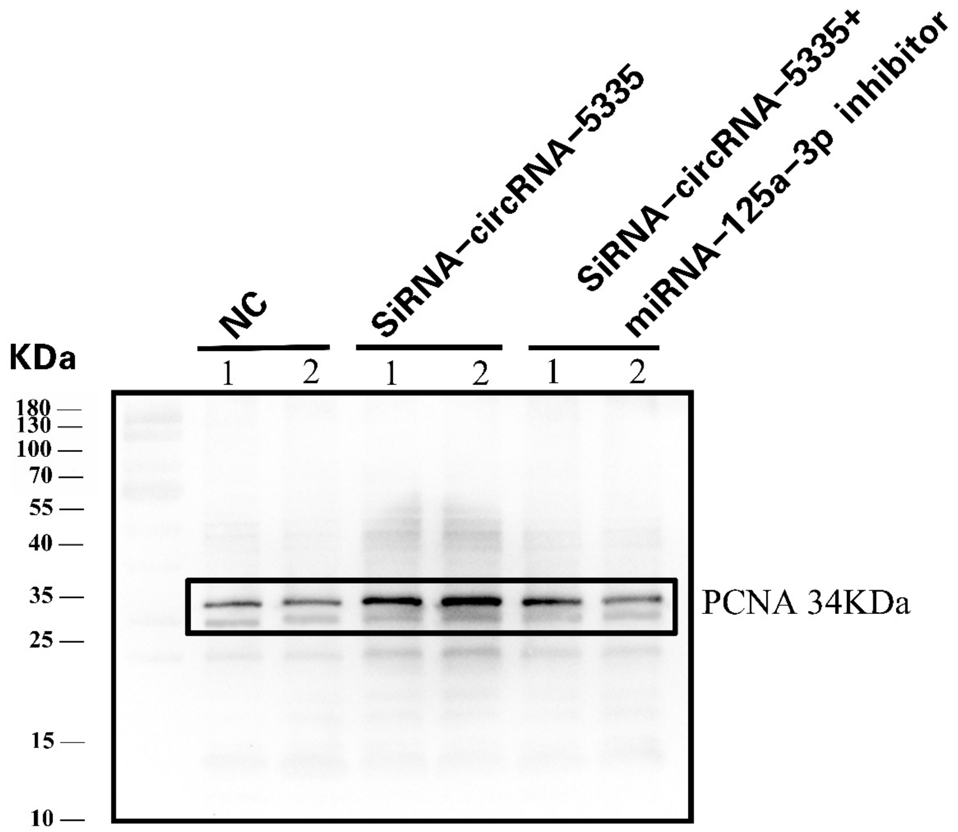
NC
SiRNA-circRNA-5335
SiRNA-circRNA-5335+
miRNA-125a-3p inhibitor
KDa
1
2
1
2
1
2
180
130
100
70
55
40
35
25
15
10
PCNA 34KDa

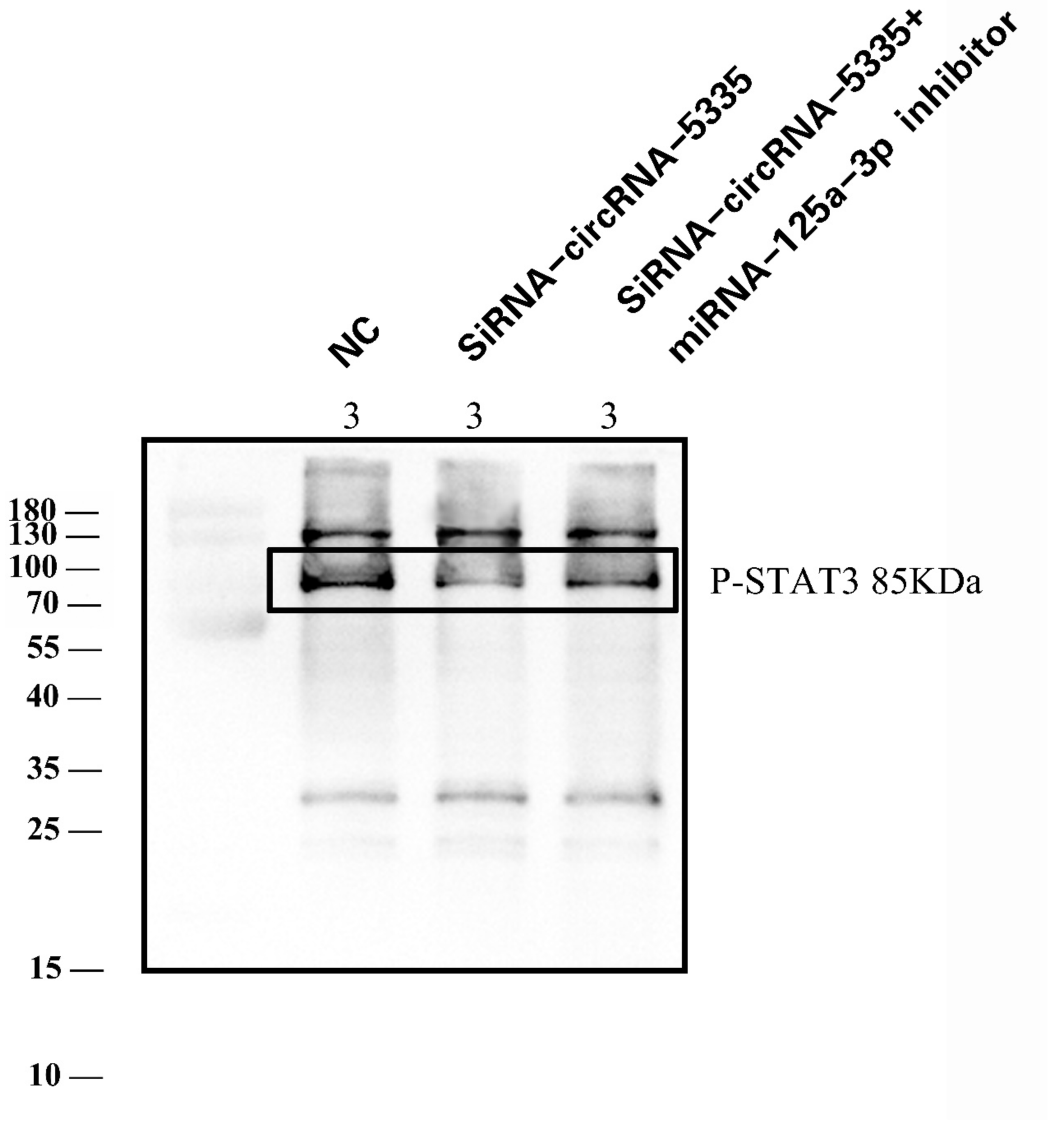

NC
SiRNA-circRNA-5335
SiRNA-circRNA-5335+
miRNA-125a-3p inhibitor
3
3
3
180
130
100
70
55
40
35
25
15
10
P-STAT3 85KDa

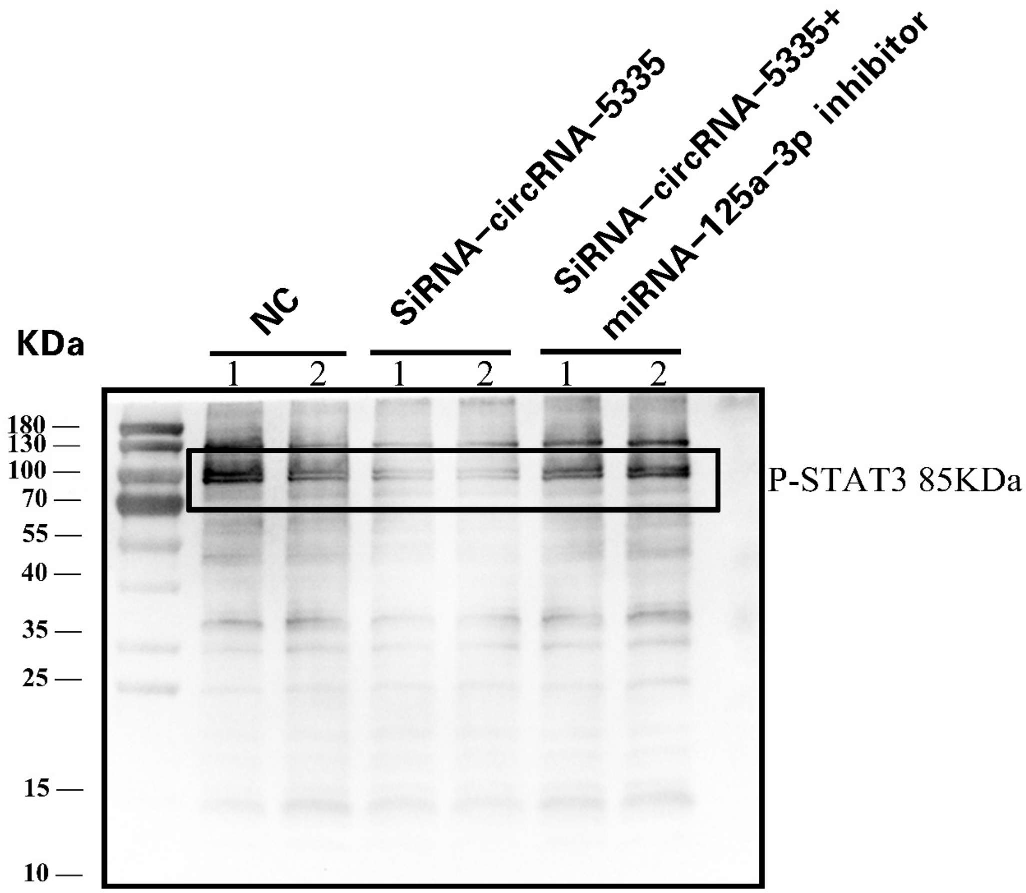
KDa
NC
SiRNA-circRNA-5335
SiRNA-circRNA-5335+
miRNA-125a-3p inhibitor
1
2
1
2
1
2
180
130
100
70
55
40
35
25
15
10
P-STAT3 85KDa

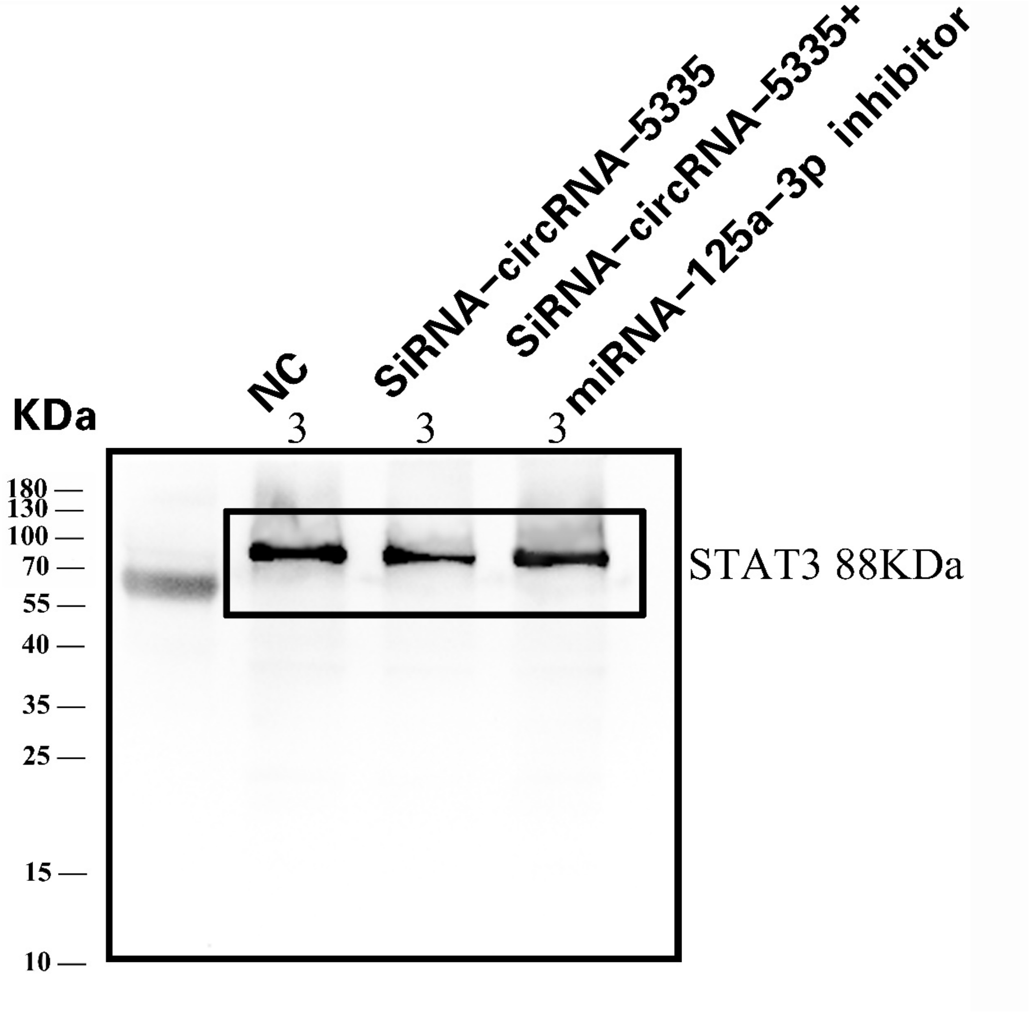

NC
SiRNA-circRNA-5335
SiRNA-circRNA-5335+ miRNA-125a-3p inhibitor
KDa
3
3
3
180
130
100
70
55
40
35
25
15
10
STAT3 88KDa

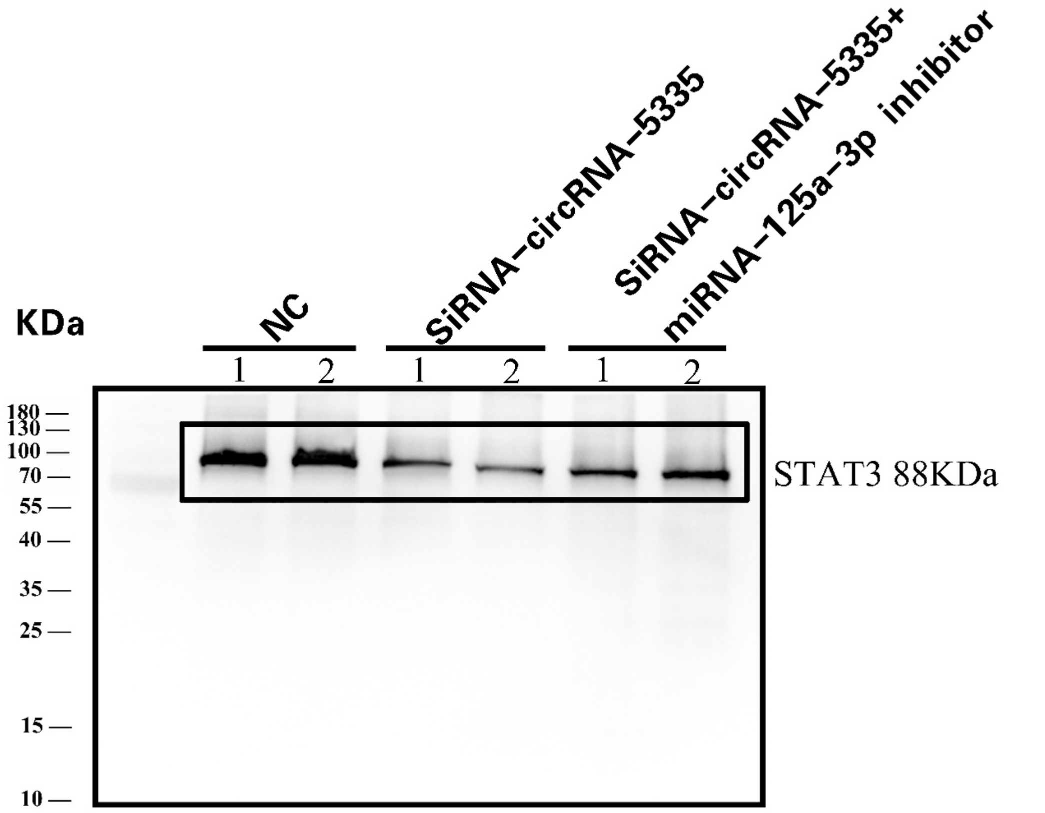

Figure S3. Alterations in the expression of GAPDH, STAT3, P-STAT3, and PCNA proteins were observed in preadipocytes treated with SiRNA-circRNA-5335 and co-transfected with miR-125a-3p or a negative control (NC) construct.

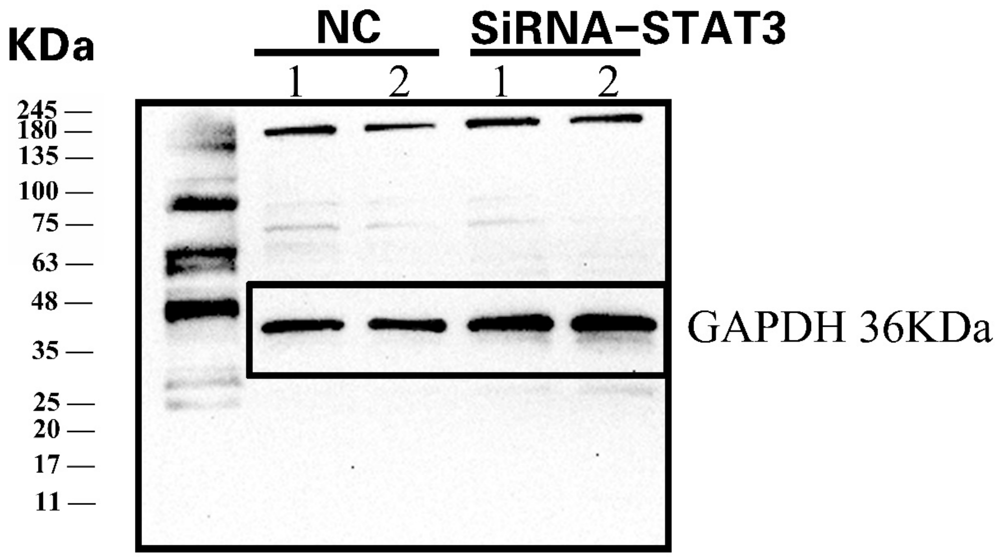

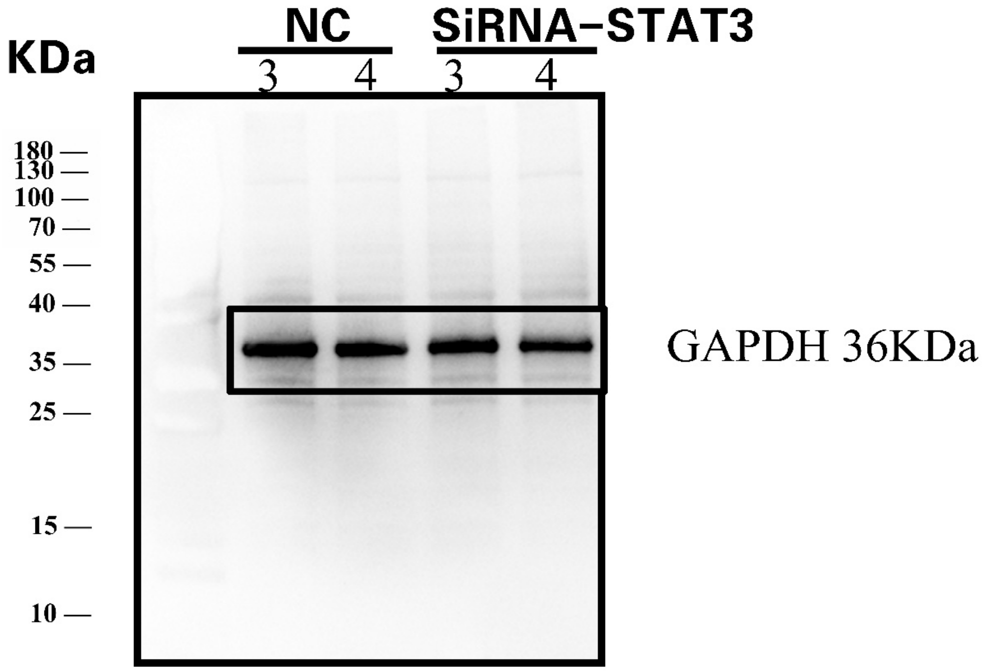
KDa
NC
SiRNA-STAT3
3
4
3
4
180
130
100
70
55
40
35
25
15
10
GAPDH 36KDa

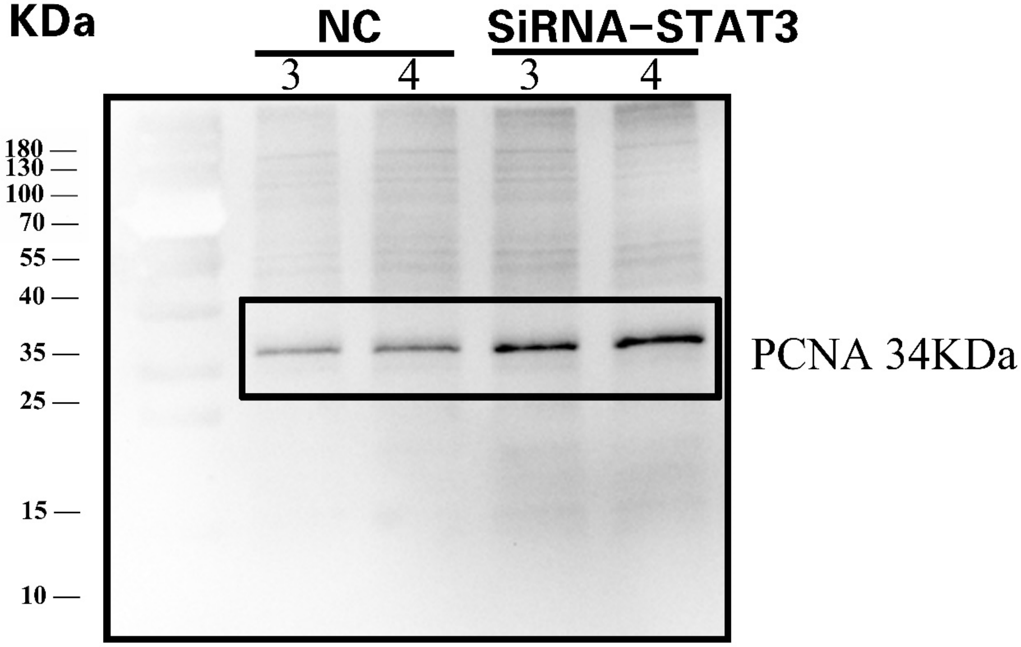
KDa
NC
SiRNA-STAT3
3
4
3
4
180
130
100
70
55
40
35
25
15
10
PCNA 34KDa

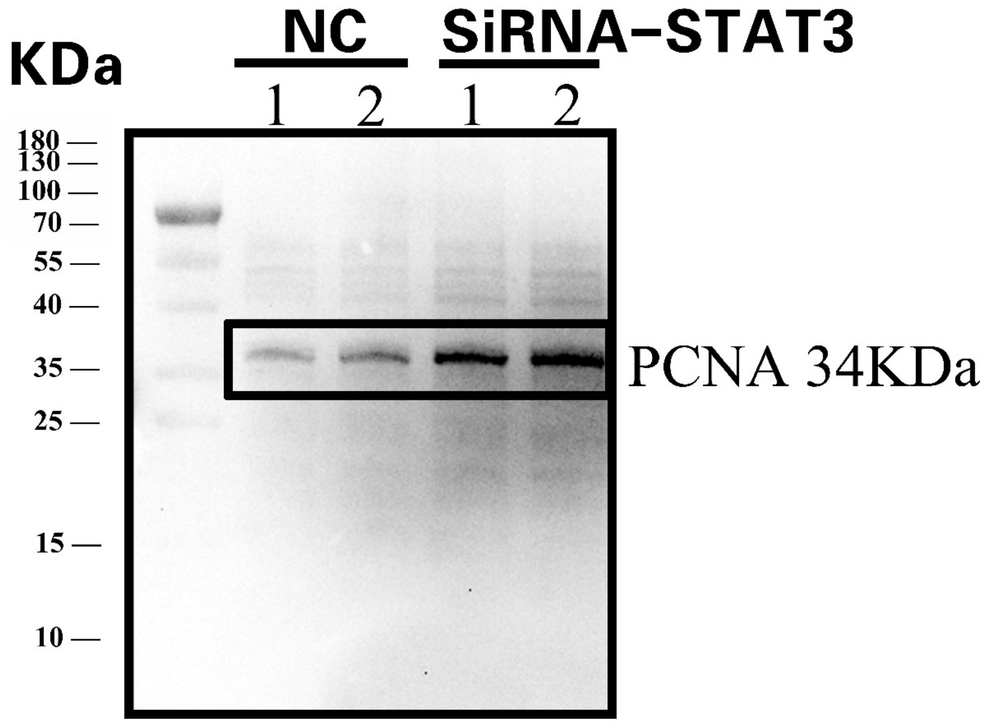
KDa
NC
SiRNA-STAT3
1
2
1
2
180
130
100
70
55
40
35
25
15
10
PCNA 34KDa

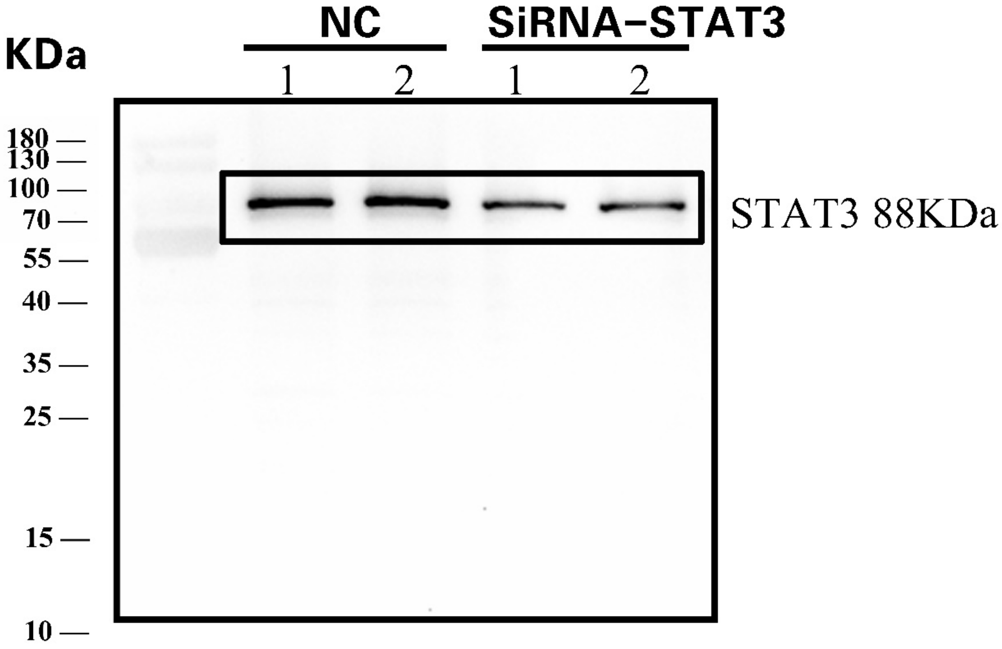
KDa
NC
SiRNA-STAT3
1
2
1
2
180
130
100
70
55
40
35
25
15
10
STAT3 88KDa

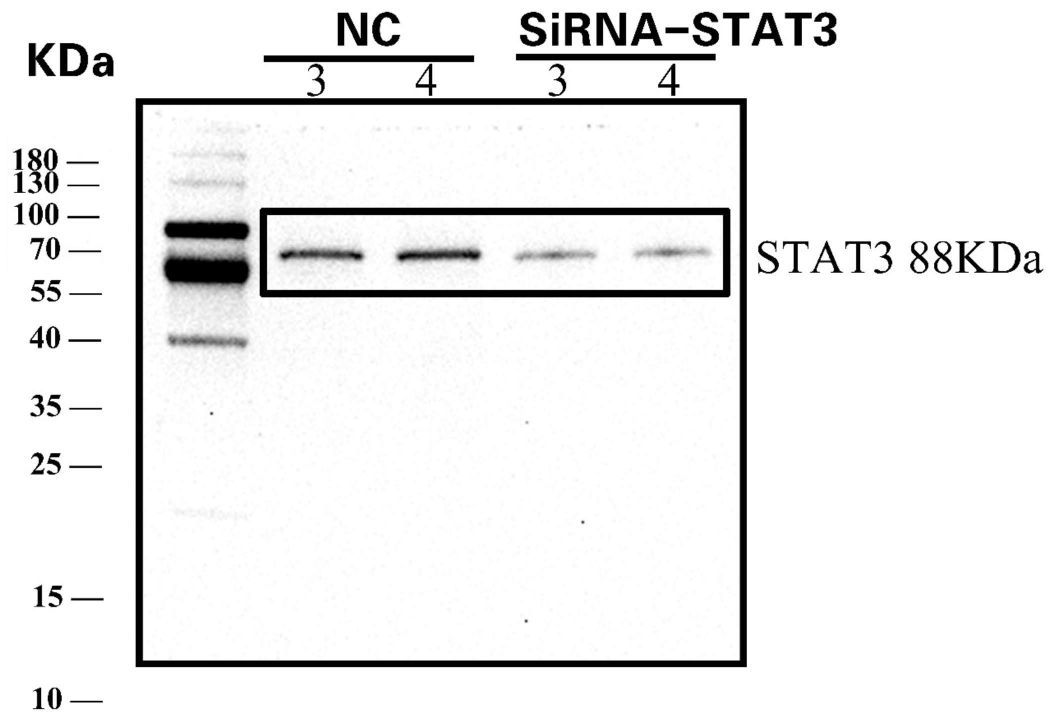

Figure S4. Alterations in the expression of GAPDH, STAT3, P-STAT3, and PCNA proteins were observed in preadipocytes treated with either a SiRNA-STAT3 or a negative control (NC) construct.
